# Sampling and processing of climate change information and disinformation across three diverse countries

**DOI:** 10.1111/bjop.70028

**Published:** 2025-09-25

**Authors:** Zahra Rahmani Azad, Tobia Spampatti, Sebastian Gluth, Kim‐Pong Tam, Ulf J. J. Hahnel

**Affiliations:** ^1^ Faculty of Psychology University of Basel Basel Switzerland; ^2^ Center for Conflict and Cooperation New York University New York USA; ^3^ Department of Psychology New York University New York USA; ^4^ Faculty of Psychology and Educational Sciences University of Geneva Geneva Switzerland; ^5^ Swiss Center for Affective Sciences University of Geneva Geneva Switzerland; ^6^ Department of Psychobiology and Methodology of Health Sciences Universitat Autònoma de Barcelona Bellaterra Spain; ^7^ Department of Psychology University of Hamburg Hamburg Germany; ^8^ The Hong Kong University of Science and Technology Hong Kong Hong Kong; ^9^ Institute for Sustainability Psychology Leuphana University Lüneburg Lüneburg Germany

**Keywords:** climate action, climate policies, confirmation bias, disinformation, information sampling

## Abstract

In the media, accurate climate information and climate disinformation often coexist and present competing narratives about climate change. Whereas previous research documented detrimental effects of disinformation on climate beliefs, little is known about how people seek climate‐related content and how this varies between cross‐cultural contexts. In a preregistered experiment, we studied how individuals sequentially sample and process Pro‐ and Anti‐climate statements across 15 rounds. Participants from the United States, China, and Germany (*N*_*total*_ = 2226) freely sampled real‐world climate‐related statements, retrieved from Twitter and validated in previous studies. Overall, reading both Pro‐ and Anti‐climate statements influenced climate concern in all countries. Participants preferred statements that were better aligned with their initial climate beliefs, and this confirmatory tendency intensified the more information had been sampled. Moreover, participants' confirmatory evaluation (i.e., accepting aligned and rejecting opposing messages) increased over time. While climate concern was mostly stable, in the United States, climate concern levels and box choices mutually reinforced each other, leading to greater polarization within the sample over the course of the experiment. The paradigm offers new perspectives on how people process and navigate conflicting narratives about climate change.

## BACKGROUND

The scientific consensus on anthropogenic climate change has been documented by the Intergovernmental Panel on Climate Change (IPCC) since its establishment in 1988 (Cook et al., 2016; IPCC, 2023; Lynas et al., 2021). For experts, the factual evidence about climate change, climate risks, and mitigation pathways is indisputable. However, for laypersons, navigating a polarized information landscape around climate change can create a different experience. On popular or social media, content about climate change is more noisy and conflicting, which may make it appear contentious or unsettled (Bolsen & Shapiro, 2018; Falkenberg et al., 2022; Lewandowsky, 2021; Petersen et al., 2019). Exposure to contrarian claims, including deliberate disinformation campaigns, can create a false perception of scientific controversy and uncertainty (Brüggemann & Engesser, 2017; Goldberg et al., 2022).

Fossil‐fuel industries have deliberately spread false claims about climate change to protect their profitable businesses for decades (Cook et al., 2019; Farrell, 2016; Oreskes & Conway, 2011; Supran et al., 2023; Supran & Oreskes, 2017). They invest heavily in campaigns designed to enhance or ‘greenwash’ their image and engage in lobbying to obstruct climate action (Brulle, 2018; Brulle et al., 2020; Brulle & Downie, 2022). Climate disinformation can be broadly categorized as either climate science denial or climate solution delay (Coan et al., 2021; Lamb et al., 2020; see also Supran & Oreskes, 2021). Climate denial disputes the scientific basis of anthropogenic climate change or the integrity of climate scientists (Dunlap & McCright, 2010). While climate delay discourses acknowledge climate change, they intentionally obstruct large‐scale climate mitigation efforts through various persuasive tactics (Doyle, 2011; Pringle & Robbins, 2022; Supran & Oreskes, 2021). Although more subtle, climate delay discourses, also known as ‘response scepticism’, are increasingly recognized as disinformation as they misrepresent robust scientific evidence about appropriate responses to climate change (Lamb et al., 2020; Painter et al., 2023; Roper et al., 2016).

Climate disinformation presents a barrier to effective climate action and policy implementation (Freelon & Wells, 2020; IPCC, 2023; Meng & Rode, 2019). For example, discussions on how to decarbonize the energy sector can get side‐tracked by false fears about wind turbines or inflated expectations about the future availability of hydrogen power (Winter et al., 2024). Common interventions to combat disinformation (Lewandowsky, 2021; Lewandowsky & van der Linden, 2021) were found to be insufficient in preventing the negative effects of repeated exposure to climate disinformation (Spampatti et al., 2024). As disinformation targets and better resonates with specific population segments, it can contribute to increasing polarization (McCright et al., 2016; Pennycook & Rand, 2021; Wojcieszak & Garrett, 2018).

People may deliberately consume disinformation as they prefer messages that confirm their existing beliefs (Jonas et al., 2001; Kerschreiter et al., 2008; Knobloch‐Westerwick & Meng, 2009) and favour news outlets that are aligned with those beliefs (Chopra et al., 2024; Garrett, 2009; Iyengar & Hahn, 2009). Confirmatory behaviour can extend beyond information selection to information processing: individuals tend to pay more attention to confirming evidence, and interpret ambiguities in ways that favour existing beliefs (Amasino et al., 2024; Corner et al., 2012; Gluth et al., 2024; Lewandowsky et al., 2012; Sunstein et al., 2016; Talluri et al., 2018). Social and cultural contexts may increase tendencies to consume belief‐confirming disinformation, as consuming news aligned with one's in‐group can serve a sense of belonging and social connection (Fielding & Hornsey, 2016; Heseltine et al., 2024).

### Cross‐cultural differences and the role of political polarization

Most research on disinformation was conducted in single Western countries, but national and cultural contexts may influence climate information search and processing (Tam et al., 2021; but see Ballew et al., 2025; Većkalov et al., 2024; Vlasceanu et al., 2024). Discourses around climate change vary between countries and differ by the degree of political polarization around climate issues (Hornsey et al., 2018; Judge et al., 2023; Lewis et al., 2019). Political ideology may serve as a lens through which climate change information is sought, filtered, and interpreted (Iyengar & Hahn, 2009; Kahan et al., 2011; Stroud, 2010). In politically polarized societies, there may be stronger tendencies to accept in‐group views, reject opposing views, and exhibit stronger selective exposure to belief‐confirming information (Bolin & Hamilton, 2018; Newman et al., 2018; West & Iyengar, 2022). Polarization of climate issues has been documented around several countries globally (Berkebile‐Weinberg et al., 2024; Pew Research Center, 2022). The partisan divide in climate views is most pronounced in the United States, where it has continuously increased over the last decades (Carmichael et al., 2017; Dunlap et al., 2016; Egan & Mullin, 2017; McCright & Dunlap, 2011), and where climate scepticism has become a characteristic stance of political conservatism (Hornsey, 2021; Kahan et al., 2011).

Compared with climate communication in the Global North, which is often characterized by pessimism, in China, optimism about climate solutions is prevalent (Graaf et al., 2024; Guenther et al., 2022; Zeng, 2022), with some potentially over‐optimistic assessments (Chu et al., 2023). China is becoming a global leader in climate action and renewable technologies, which contributes to national pride and support for governmental climate initiatives (Guo et al., 2023). Generally, there is a high awareness of anthropogenic climate change concern in China (Lewis et al., 2019; Wang & Zhou, 2020), but anti‐Western conspiracy beliefs against climate action also seem to exist (Du et al., 2024; Liu, 2015, 2023; Tam & Chan, 2023). Comparing information search of individuals from countries with various climate discourses can contribute to a better understanding of how climate beliefs form within a population and help to improve context‐specific climate communication.

### The present study

The findings on the detrimental effects of climate disinformation underscore the need to examine how individuals actively seek climate information. Understanding what predicts selection and integration of either accurate information or disinformation can serve as a starting point for effective strategies to curb exposure to disinformation. In this preregistered study, we employ a novel sequential sampling paradigm to investigate how individuals select and process contradictory climate‐related statements. Over multiple rounds, participants freely chose between two boxes featuring contrasting climate content. After every box choice, participants were asked to rate how much they agreed with the statement featured by the box and their momentary climate change concern. The ‘Anti‐climate box’ contained climate disinformation, including statements that questioned climate science or argued against climate action, reflecting climate denial or climate delay narratives as described above. In contrast, the ‘Pro‐climate box’ featured statements advocating for decisive climate action and presenting findings from climate science. All statements were empirically pretested, real‐world social media posts collected from the platform X/Twitter, sourced from a diverse array of accounts across more than 19 countries (Spampatti et al., 2025).

Our experimental paradigm allows us to test for confirmatory behaviour both on the level of source selection and content evaluation. We expected climate change beliefs measured at the beginning of the study to predict box choices and statement evaluations. Specifically, we hypothesized that people with higher initial climate beliefs select the Pro‐climate box more often and agree with its statements more compared with people with lower initial beliefs. Because of different levels of political polarization, we expected these effects to be stronger in the United States than in China and Germany (Hypothesis 1). Second, we expected momentary climate change concern to increase after choosing a statement from the Pro‐climate box and to decrease after reading an Anti‐climate statement (Hypothesis 2). Taken together, the preference for belief‐confirming information and the persuasive impact of messages can lead to reciprocal effects that mutually reinforce each other. The sampling might bolster existing attitudes, such that the views of climate sceptics and believers become more extreme over time, potentially leading to their divergence. The reinforcing spirals model posits that under some circumstances, positive feedback loops can occur, where views become more polarized (Slater, 2007; Spohr, 2017). We therefore expected that climate change concern increases during the sampling task in participants who selected the Pro‐climate box more often and decreases in participants who selected it less (Hypothesis 3).

## METHOD

### Participants

The data, and code are available on OSF (https://osf.io/w4kdh). In the United States and Germany, data were collected via a market research institute as part of a larger panel study. The panel study included two other experimental tasks for separate research projects that are not reported here. The order of the three tasks was randomized and we controlled for the order of the experimental sampling tasks in the subsequent analyses (i.e., whether the sampling task was completed in first, second, or third position). In China, we collected data separately with a different market research institute. We preregistered a sample size of *N* = 3000 participants (*n* = 1000 per country).

We aimed to obtain nationally representative samples, with simple quotas for age, gender, income and residential area (urban/rural) in the United States and Germany. In China, we could apply simple quotas for age and gender only. Due to difficulties with data collection and budget constraints, particularly in the larger panel study, the actual sample sizes deviated from the preregistered target sizes. We obtained data from *n* = 880 participants in the United States, *n* = 883 in Germany and *n* = 1136 in China. We applied two post hoc exclusion criteria to ensure high data quality and to reduce the likelihood of data points based on insincere participation (Goodrich et al., 2023; Storozuk et al., 2020). These exclusion criteria were (1) following instructions to move range sliders to the leftmost position after round 7, and (2) sampling from either of the two boxes at least once. Since participants received no prior information about the boxes, they could only learn about them through sampling. Participants who failed one of these attention checks were excluded. Analyses conducted on the full sample (see Supporting Information) yielded consistent results, confirming the findings reported in the main manuscript. This procedure differs from the preregistration where we stated to report the results based on all participants in the main manuscript and the robustness analysis with attentive participants in the Supporting Information. We opted for this procedure because the number of inattentive participants was larger than expected. This was partly attributable to a technical failure in a prestudy screening filter, which was designed to prevent automated bots and inattentive users from entering the study. The final sample sizes were *n* = 674 participants from the United States, *n* = 795 from Germany and *n* = 757 from China. We report the demographic composition of the samples in the Tables S1–S4.

### Materials

#### Sampling task

**FIGURE 1:**
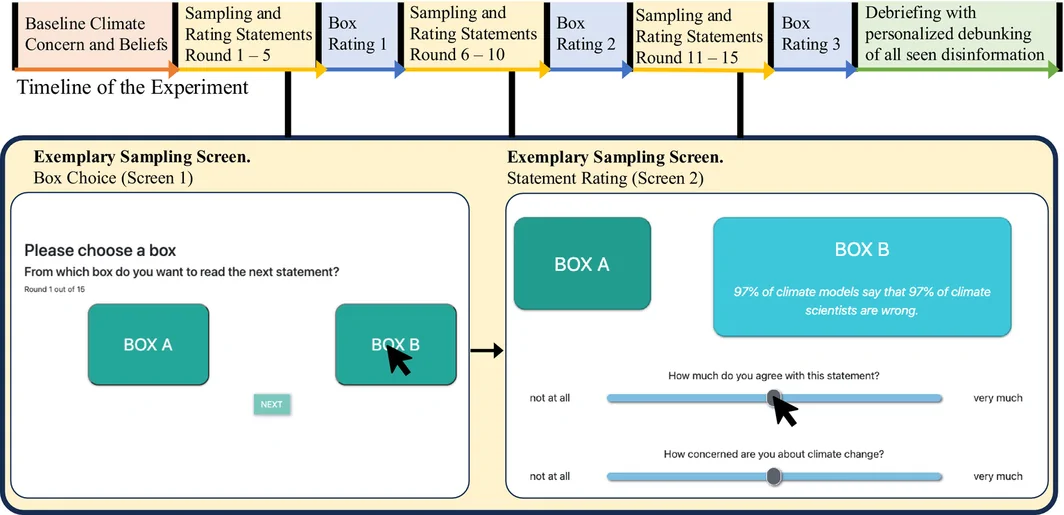
Procedure of the experiment with 15 rounds of statement sampling and rating.

Over 15 rounds, participants repeatedly chose between two boxes (Figure 1). One box consistently revealed a Pro‐climate statement, while the other box consistently revealed an Anti‐climate statement. Participants then rated how much they agreed with the statement and indicated their momentary climate change concern on range sliders. Whether Box A (left‐hand side) or Box B (right‐hand side) was the Pro‐ or Anti‐climate box was counterbalanced between participants. There was no a priori information about the content of the boxes, and participants could only learn about them through exploration. A pretest (*n* = 500 from the United Kingdom) compared informative box labels (‘Pro‐climate’ box and ‘Anti‐climate’ box) with neutral box labels (‘Box A’ and ‘Box B’). In this pretest, no differences in the distribution of box choices were found between the label conditions.

In every round, participants could freely choose between one of the two boxes to retrieve a randomly drawn statement from the respective Pro‐climate or Anti‐climate database. Statements were drawn without replacement for each participant to avoid repetition. Every fifth round, participants were asked to rate the boxes. They indicated their liking for Box A and Box B on one‐item measures, and whether they would follow an account with similar content on social media (see Figure S3).

#### *Real‐world* stimuli used for the sampling task

The statements used in the sampling task were derived from two databases with real‐world climate‐related statements from public Twitter (now X) posts. Every statement had previously been manually coded and empirically tested in validation studies. The ‘Anti‐climate’ database featured 75 disinformation statements downplaying and contesting climate change (Spampatti et al., 2025). To retrieve eligible statements, a list with Twitter accounts from conservative think‐tanks, members of the climate‐counter movement, and climate‐denying institutions was curated. All public posts by these accounts (until October 2022) were downloaded and a random subset of 20,000 posts was manually coded. Manual coding identified climate‐relevant posts that were understandable with minimal background knowledge. All eligible statements were classified among a taxonomy for Anti‐climate arguments based on Lamb et al. (2020) and Coan et al. (2021). The remaining 78 statements were empirically tested with a representative sample from the UK (with *N* = 500). In the validation study, participants rated the statements on various dimensions (e.g., whether they were familiar with it, its persuasiveness, political slant or emotional content). Every statement was rated by 50–70 participants. For the present study, we excluded three items because they contained slurs against China.

The ‘Pro‐climate’ database contained 146 statements that promoted facts about the severity of climate change and the urgency of climate action. It was developed similarly to the Anti‐climate database. A list of Twitter accounts from climate scientists, NGOs, climate activists, and science journalists from more than 19 different countries was curated. All their public posts in the English language (until January 2023) were scraped from Twitter, totalling 4.3 million posts. A randomly drawn subset of 20,000 posts was manually coded, and if applicable, classified among a taxonomy for Pro‐climate communication. Similarly to the Anti‐climate database, the resulting 146 statements were empirically tested in a validation study with a representative sample from the UK with *N* = 500. All stimuli were originally posted in English. A professional translation agency translated and back‐translated all statements to simplified Chinese, and we carefully checked for discrepancies in the back‐translations. Additionally, four native Chinese speakers also reviewed the translations of all statements and all instruction texts for understandability and idiomatic language use, and we revised the Chinese text according to their suggestions. Native German speakers independently translated and back‐translated the statements to German.

For both databases, two independent raters carefully fact‐checked every statement against the best available evidence. All statements from the Anti‐climate database were identified as inaccurate contrarian claims, reflecting climate denial and delay narratives. While the Pro‐climate statements were largely accurate information about the risks of climate change and the benefits of climate mitigation measures, we identified nine inaccurate ones. For every inaccurate statement, debunking corrections were formulated with scientific references. At the end of the study, all participants read a note about the scientific consensus on climate change (with country‐specific links to additional official information about climate change). Additionally, participants received corrections for every false or misleading statement they had encountered during the sampling task. This in‐depth debriefing was employed to mitigate the harm of disinformation exposure. The procedure was approved by Ethics Committee of the Faculty of Psychology at the University of Basel (007‐23‐1).

#### Climate belief questionnaire

To assess climate change beliefs, we used a six‐item scale from van Valkengoed et al. (2021). We used two subscales (belief in anthropogenic climate change and the severity of the consequences of climate change) with three items each (e.g., ‘*Climate change will bring about serious negative consequences*’; see Figure S1 for full scale). Agreement to these statements was measured on 10‐point Likert scales (from ‘*not at all*’ to ‘*fully*’). Participants scoring low on these scales can be classified as *climate sceptics* who are not convinced that human activities cause climate change with serious negative consequences. High scorers are often referred to as *climate believers*, implying that they acknowledge the human causation and severity of climate change.^1^ Internal consistency of the joint scale was Cronbach's alpha = .88 which was higher than for either subscale alone (alpha = .79 and alpha = .83). We therefore aggregated all items to a score assessing initial climate change beliefs.

### Procedure

An illustration of the procedure can be found in Figure 1. After providing informed consent, participants rated their climate change concern on a continuous range slider (from *not at all* = 0 to *very much* = 100) and filled out the climate beliefs questionnaire as a measure of baseline attitudes about climate change. Participants then proceeded to the instructions explaining that they could freely sample statements as they would when browsing information online. We intentionally did not provide more specific instructions about how they should sample statements because we were interested in their spontaneous sampling behaviour.

Participants completed 15 rounds of sampling climate‐related statements from two boxes as described above. In the end, they were debriefed with general information about the scientific consensus on climate change and received corrections for every piece of misinformation they had seen.

### Data analysis

Statistical analyses were conducted using R version 4.3.3 (R Core Team, 2020) and several software packages: lme4 (Bates et al., 2015), lmerTest (Kuznetsova et al., 2017), lavaan (Rosseel, 2012) and tidyverse (Wickham et al., 2019). Whenever we report additional non‐preregistered analyses, we label them as exploratory (Preregistration: https://osf.io/hr4pb). In all regression models, wherever applicable, we controlled for the effects of order of the task, country, and round number. We tested the following hypotheses:

We hypothesized that participants would prefer the belief‐congruent box. Specifically, we expected participants with stronger climate beliefs to choose the Pro‐climate box and those with climate‐sceptical beliefs to choose the Anti‐climate box more often.

We hypothesized that this relationship would be stronger in the United States than in China or Germany.

We tested if the preference for the belief‐congruent box would be more pronounced in later rounds.

For Hypothesis 1, we computed a mixed‐effects logistic regression model with random intercepts for participants and box choice as a dependent variable. In the preregistration, we erroneously indicated including random intercepts for statements, which, however, would be a misspecification of the model because they perfectly correspond with the dependent variable of box choice. Fixed effects were initial climate beliefs. For H1b, in a second step, we computed a model that additionally included the interaction of initial climate beliefs by country to test for differences between countries. For H1c, we added the interaction between initial climate beliefs and round number to the logistic regression.

We expected that higher [lower] initial climate beliefs would predict higher agreement with Pro‐climate statements [with Anti‐climate statements].

We expected that the relationship between initial climate beliefs and statement agreement by statement type would be stronger in the United States compared with China and Germany. To test H1d, we computed a linear mixed‐effect regression model with agreement to statement as the dependent variable. As fixed effects, we included initial climate beliefs, statement type (Pro‐ vs. Anti‐climate statement), and their interaction. As random effects, we included participant‐ID and statement‐ID. Exploratively, we also included the three‐way interaction with round number to test for temporal effects in statement evaluation. For H1e, we added the three‐way interaction with country to test for country‐specific differences in the hypothesized relationship.

We expected that box choices would correspond to variations in individual climate change concern, with higher [lower] climate concern immediately after reading a Pro [Anti] climate action statement.

Further, we expected that this effect would be moderated by agreement with the statement. This means the more a participant agrees with a statement, the more influential the statement is on the participant's climate change concern level.

We tested if the effect of box choice on momentary climate change concern differed between countries.

We tested H2a with a mixed‐effects linear regression model with random intercepts for participants for statement, and climate change concern as a dependent variable. Fixed effects were selected box type and initial climate change concern. For H2b, we added the interaction with statement agreement. For H2c, we added the interaction with country to test if the effects of box choice on climate concern varied between countries. To further examine and visualize this effect, we conducted a robustness check using within‐person centred climate change concern. Within‐person centring captured roundwise variation of a participant's average concern over all 16 measurement points. We then tested whether these roundwise variations in climate change concern differed after reading a Pro‐ or Anti‐climate statement.

We expected the number of Pro‐climate box choices to be predictive for individual changes in climate concern over the course of 15 rounds. To test H3, we first computed random effects only models with climate change concern as a dependent variable, and random intercepts and random slopes by participant for round number. From these models, we extracted individual slopes in climate change concern for every participant. For each country, we then regressed the individual slopes onto the total number of Pro‐climate box choices while controlling for initial climate concern. As an additional exploratory robustness check for H3, we also fitted a latent growth curve model to test whether the total number of box choices could significantly predict the change in climate concern over the course of the 15 rounds.

Additionally, we preregistered a number of secondary hypotheses that are reported in detail in the Supporting Information. We had also preregistered additional analyses that include political worldviews as a predictor variable. Since we were not able to assess these worldview measures in China, we report these analyses for the Western countries only.

## RESULTS

### Distribution of the main dependent variables

#### Climate change concern

Initial climate change concern was above the midpoint in all countries. In China, average initial climate change concern (M = 75.24; 95% CI [73.96; 76.52]) was significantly higher than in the United States (M = 67.34; 95% CI [64.91; 69.78]) and Germany (M = 64.21; 95% CI [62.08; 66.35]). Initial climate change concern in China was almost uniformly high, whereas responses in Western countries were more varied, with a greater concentration at the extremes. This was also reflected in the bimodality coefficients, a measure to assess whether a distribution has two rather than one peaks and which is also used as an indication for polarization of a sample (Fischer & Frey, 2023). Bimodality coefficients for initial climate change concern were above the threshold of 0.555 (Freeman & Dale, 2013) in the Western countries (US = .70, Germany = .65) suggesting a bimodal distribution and below in China (0.41), consistent with a unimodal distribution. The Figure S4 displays the distribution of initial climate change concern.

#### Box choices

The distribution of box choices across all participants and rounds was relatively even, with 53.1% of all selections made for the Pro‐climate box. The mean number of Pro‐climate box choices was slightly but significantly higher than the midpoint of 7.5 in all countries. This indicates that, in aggregate, box choices were relatively evenly distributed, with a slight overall preference for the Pro‐climate box. The average number of Pro‐climate box choices was 7.86 in China (95% CI [7.63, 8.09]), 7.99 in the United States (95% CI [7.82, 8.15]), and 8.04 in Germany (95% CI [7.87, 8.21]). The between‐participant standard deviation in box choices was greater in China (SD = 3.21) than in the United States (SD = 2.21) and Germany (SD = 2.39), as indicated by Levene's test for equality of variances, *F*(1, 2224) = 112.9, *p* < .001. This larger variance in China indicates greater heterogeneity in individual choice patterns. Specifically, a greater proportion of Chinese participants made choices favouring either the Pro‐ or Anti‐climate box, while participants in the Western countries tended to sample more evenly. Plots displaying the distribution of box choices can be found in the Figure S5.

### Predicting box selection from initial climate beliefs

**FIGURE 2:**
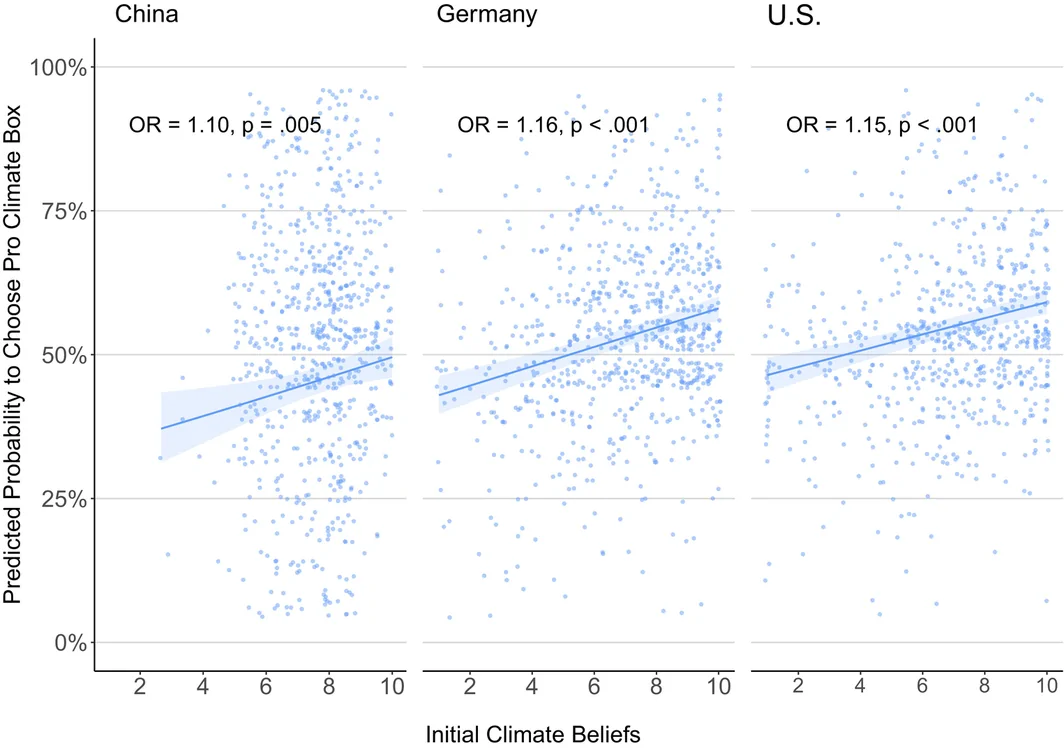
The relationship between initial climate beliefs and box choices in the three countries. *Note*: The slopes show the predicted probabilities to choose the Pro‐climate box from the mixed‐effect logistic regression models; the points show the actual percentage of Pro‐climate box choices by participants. Shaded areas show the 95% confidence interval for the predicted values of the regression model.

We hypothesized (H1) that participants select the belief‐congruent box more often, meaning that initial climate beliefs can predict box choices in the sampling task. The logistic mixed‐effects regression model revealed a significant main effect of initial beliefs on box choice (OR_initialBeliefs_ = 1.14, 95% CI [1.11, 1.18], *p* < .001). In line with preregistered H1a, higher initial climate beliefs were associated with a greater propensity to select the Pro‐climate box (see Figure 2). Moreover, there was a main effect of round number on box choices, indicating that in later rounds, the likelihood to choose the Pro‐climate box increased (OR_roundNumber_ = 1.09, 95% CI [1.07, 1.12], *p* < .001).

Table 1 presents the results of the logistic mixed‐effects models computed separately for each country. Descriptively, the confirmation bias effect was stronger in the Western countries than in China. Contrary to H1b, we did not find statistically significant differences in the strength of confirmation bias between countries, as indicated by a non‐significant interaction of country and initial climate beliefs (OR_ChinaVSGermany_ = 1.03, 95% CI [0.94, 1.13], *p* = .565, OR_ChinaVsUSA_ = 1.00, 95% CI [0.91, 1.10], *p* = .954).

**TABLE 1:** Logistic mixed‐effect models predicting box choice from initial beliefs and round number.

Predictors	China			USA			Germany		
	Odds ratios	95% CI	*p*	Odds ratios	95% CI	*p*	Odds ratios	95% CI	*p*
(Intercept)	1.52	1.38–1.67	**<0.001**	1.20	1.07–1.35	**<0.001**	1.18	1.03–1.34	**<0.001**
Initial beliefs	1.10	1.03–1.17	**0.005**	1.15	1.10–1.20	**<0.001**	1.16	1.11–1.22	**<0.001**
Round number	1.13	1.08–1.17	**<0.001**	1.05	1.01–1.09	**0.022**	1.10	1.06–1.14	**<0.001**
Initial Beliefs × round number	1.06	1.02–1.10	**0.006**	1.05	1.01–1.09	**0.017**	1.08	1.04–1.12	**<0.001**
Box A is Pro‐climate	0.55	0.48–0.63	**<0.001**	0.89	0.82–0.98	**0.012**	0.93	0.85–1.02	0.115
Position Sampling Task				1.00	0.95–1.05	0.931	1.01	0.96–1.07	0.639
Random effects									
σ^2^	3.29			3.29			3.29		
τ_00_	0.55_participant‐ID_			0.07_participant‐ID_			0.14_participant‐ID_		
ICC	0.14			0.02			0.04		
N	757_participant‐ID_			674_participant‐ID_			795_participant‐ID_		
Observations	11,355			10,110			11,925		

*Note*: Regression models separate by country for the effect of initial climate beliefs and round number on box choice. Counterbalancing (whether Box A was the Pro‐ or Anti‐climate box) and position/order of the sampling task within the larger panel study (not relevant for China, where data were collected independently) were added as covariates. Bold indicates statistically significant values at the α = .05 level.

**FIGURE 3:**
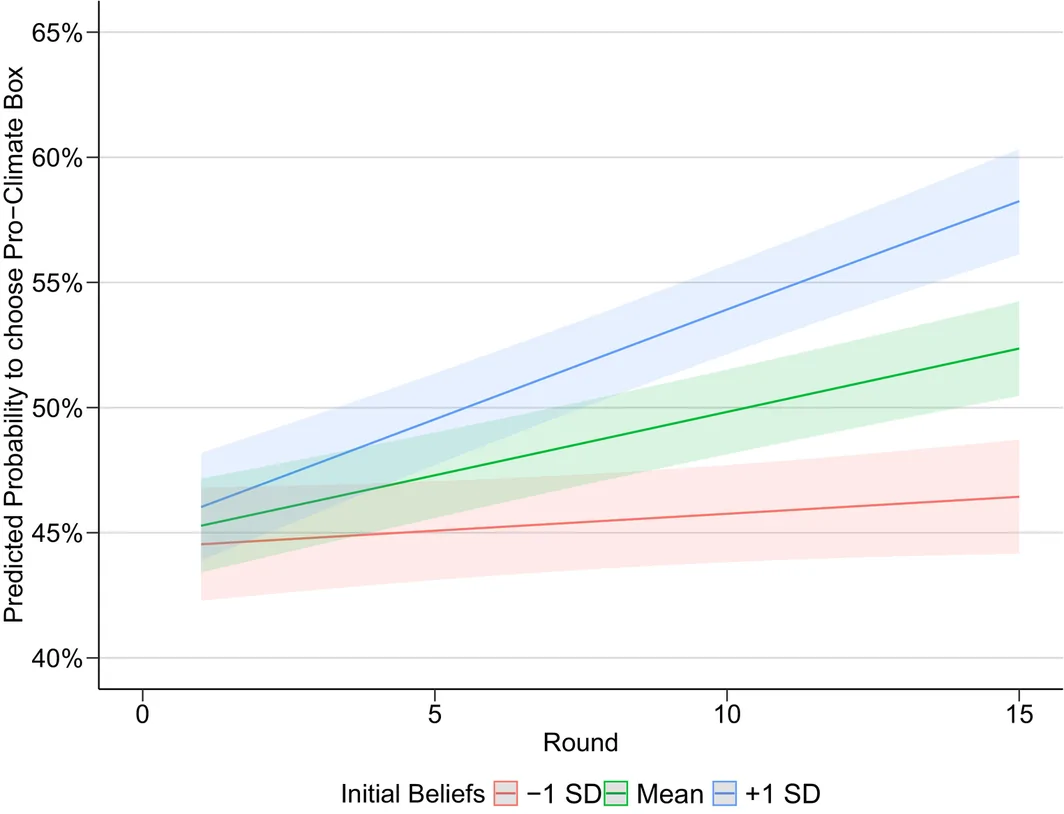
The effect of initial beliefs on box choices becomes more pronounced with increasing round number. *Note*: The lines show how the predicted probability of choosing the Pro‐climate box changes over the course of rounds, depending on participants' initial climate beliefs. Shaded areas show the 95% confidence intervals for predicted values of the regression model.

To test explorative H1c, we added the interaction between initial climate beliefs and round number, which was significant (OR_interaction_ = 1.07, 95% CI [1.04, 1.09], *p* < .001). This indicated that the effect of climate beliefs on box choices was stronger in later rounds (see Figure 3). As one might contend that this interaction can be attributed to the fact that participants needed to explore the boxes first before developing a preference, we tested if the interaction effect remained significant after removing the first five rounds. A logistic mixed‐effects model with the same specification including only rounds 6–15 revealed a smaller, but still significant effect for the interaction of round number and initial beliefs (OR_interaction_ = 1.04, 95% CI [1.02, 1.07], *p* = .002). This suggests that even after participants formed initial impressions of the boxes, participants preferred the more belief‐congruent box the more they had sampled.

### Predicting agreement to statement by initial beliefs

Using mixed‐effects regression models, we tested preregistered H1d that agreement to a statement type (Pro‐ vs. Anti‐Climate) could be predicted from initial climate beliefs. Indeed, the interaction between statement type and initial climate beliefs was significant (β_Anti‐ClimateXInitialBeliefs_ = −23.29, 95% CI [−23.81; −22.76], *p* < .001). This indicates that participants with higher climate beliefs agreed with Anti‐climate statements substantially less than participants with lower initial climate beliefs (see Figure S8 for a visualization). Looking at the temporal component, we found a significant three‐way interaction with round number (β_Anti‐ClimateXInitialBeliefsXRound_ = −1.78, 95% CI [−2.30 – −1.27], *p* < .001). This indicates that over the course of the sampling task, the evaluations of the statements became more slanted towards preferring confirmatory statements. Again, testing for differences between countries, we added the three‐way interaction with country to the regression model. This interaction was significant, suggesting that confirmatory statement evaluation, while significant in all countries, was higher in the United States (β_Anti‐ClimateXInitialBeliefsChinaVsUS_ = −8.61, 95% CI [−10.22; −7.00], *p* < .001) and in Germany (β_Anti‐ClimateXInitialBeliefsChinaVsGer_ = −10.64, 95% CI [−12.25; −9.03], *p* < .001) compared with China.

### Effects of climate information and disinformation on climate concern

Next, we tested preregistered H2a that reading a Pro‐ or Anti‐climate statement affected momentary climate change concern. As expected, we found a significant effect of box choice on climate change concern on the trial level (see Table 2) indicating that climate concern levels decreased after reading an Anti‐climate statement and increased after reading a Pro‐climate statement compared with a participant's initial level of concern. Supporting preregistered H2b, there was a significant interaction between agreement to the statement and statement type on climate change concern (β = −1.84, 95% CI [−2.10; −1.59], *p* < .001), indicating that higher agreement to a statement was associated with the statement having stronger effects on climate change concern. In other words, the content of the statement (Pro‐ or Anti‐climate) was associated with fluctuations in participants' climate concern, and more so, the more they agreed with the statement. To test the explorative H2c, if the effect of box choice on climate concern differed between countries, we examined the interaction with country. Indeed, the effect of box choice was stronger in China than in the Western countries, as indicated in significant interaction effects (China vs. US: β = 0.88, 95% CI [0.31; 1.46], *p* = .002; China vs. Germany: β = 0.89, 95% CI [0.34; 1.45], *p* = .002). In the individual country models, the effect remained significant in all three countries at the *p* < .001 level; China, however, showed the strongest effect.

**TABLE 2:** Results of a mixed‐effects regression model of the effect of box choice on individual‐level climate change concern.

Predictors	Roundwise climate concern		
	Estimates	CI	*p*
(Intercept)	7.57	5.74–9.40	**<0.001**
Box choice [Anti‐climate]	−1.29	−1.52 – −1.06	**<0.001**
Initial Climate Concern	0.89	0.87–0.91	**<0.001**
Country [Germany]	0.89	−0.47 – 2.25	0.198
Country [USA]	0.33	−1.12 – 1.77	0.658
Round number	0.00	−0.02 – 0.03	0.833
Position Sampling Task	0.10	−0.61 – 0.80	0.787
Box A is Pro‐climate	1.23	0.26–2.21	**0.013**
**Random effects**			
σ^2^	91.24		
τ_00 participant‐ID_	131.57		
τ_00 statement‐ID_	0.27		
ICC	0.59		
N_participant‐ID_	2226		
N_statement‐ID_	219		
Observations	33,390		

*Note*: Regression Model predicting variation in climate change concern after reading a Pro‐ or Anti‐ climate statement controlling for initial climate concern. The model controls for country, round number, counterbalancing (whether Box A was the Pro‐ or Anti‐climate box) and position/order of the sampling task within the larger panel study (not relevant for China, where data was collected independently). Bold indicates statistically significant values at the α = .05 level.

**FIGURE 4:**
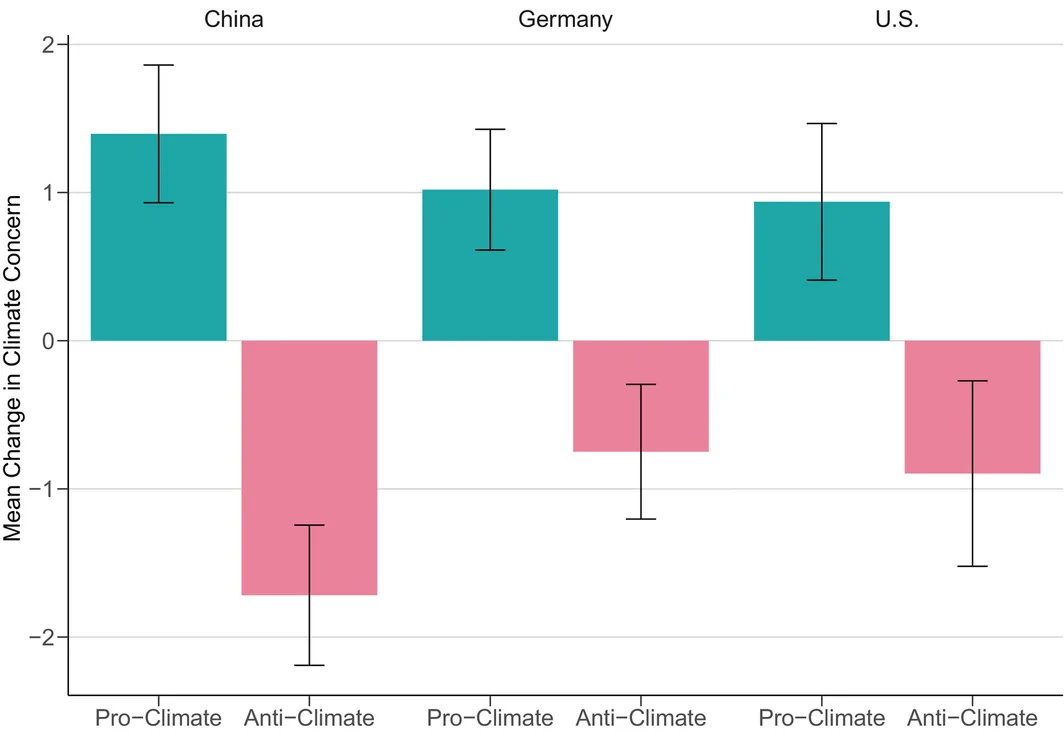
Mean change in within‐person centred climate change concern after reading a Pro‐ or Anti‐climate statement. *Note*: The y‐axis displays unstandardized deviations from a person's mean climate concern (= zero) after reading a Pro‐ or Anti‐climate statement. The error bars represent the 95% confidence interval of the mean.

In our robustness check for H2a, we examined the deviations in climate change concern from participants' average levels of concern after each round. The results by country are depicted in Figure 4: After reading an Anti‐climate statement, within‐person centred climate change concern was significantly below zero in all countries (i.e., lower than the average climate concern of a participant). Similarly, climate change concern was significantly above a participant's average levels after reading a Pro‐climate statement in all countries. This suggests that exposure to a Pro‐ or Anti‐climate statement predicted individual variations in climate concern.

### Reinforcing spiral between climate concern and information sampling

**FIGURE 5:**
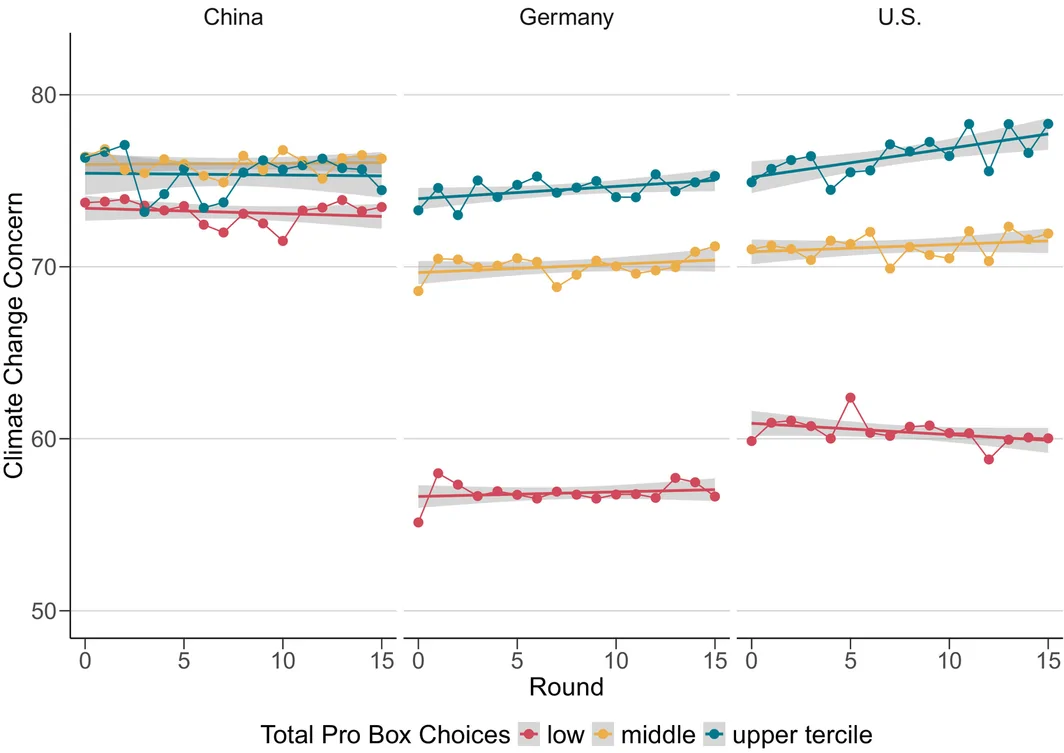
The evolution of climate change concern (measured from 0 to 100) across the sampling task. *Note*: The evolution of climate change concern across 15 rounds of choosing between Pro and Anti‐climate statements is shown for participants in the upper, middle, or lower tercile for total Pro‐climate box choices. The shaded areas show the 95% confidence interval for the smoothed regression line based on a linear model. Round 0 indicates initial climate change concern before the sampling task.

To examine preregistered H3, we investigated whether the evolution of climate change concern varied as a function of participants' sampling patterns. Hypothesis 3 predicts a cumulative effect: multiple Pro‐climate box choices should lead to a steady increase in climate change concern. Figure 5 displays trajectories of climate change concern for participants grouped into terciles based on the frequency of Pro‐climate box choices (many, medium and few). First, it can be seen that the amount of box choices differentiates between different levels of climate concern in the Western countries and, to a much lesser degree, in China. In the United States and Germany, initial climate beliefs strongly corresponded with patterns of box choices: the tercile of participants who selected the Pro‐climate box most often had higher levels of climate beliefs than participants with Pro‐climate box choices in the lowest tercile. In China, participants with different sampling patterns hardly differed in their climate beliefs. Second, it also shows how climate change concern changes throughout the experiment for participants with different box preferences. For the United States, visual inspection suggests reinforcing effects, where participants' initial climate change concern drifts towards more extreme points alongside belief‐confirming information search. That is, the sampling task led to an increase in the gap between participants expressing high and low climate change concern.

To test this hypothesis statistically, we regressed individual slopes in climate concern onto the number of Pro‐climate box choices while controlling for individual differences in initial climate change concern. In the United States, but not in the other countries, the number of Pro‐climate box choices significantly predicted individual slopes in climate change concern, β = 0.23, 95% CI [0.06; 0.39], *p* = .008 (regression coefficients for China β = 0.00, 95% CI [−0.08; 0.08], *p* = .954 and for Germany β = 0.09, 95% CI [−0.03; 0.21], *p* = .142). This indicates that slopes of climate change concern were increasing more in US‐American participants with higher amounts of Pro‐climate box choices and decreasing with lower amounts of Pro‐climate box choices. In the United States, our data and statistical results are consistent with reinforcing spiral models. These models posit that the interplay between climate concern levels and information search can intensify high or low levels of climate concern (Slater, 2007). Consequently, this can lead to an increasing divergence between different segments of the sample.

As an additional, explorative test of H3, we fitted a latent growth curve model to examine the effect of Pro‐climate box choices on change in climate concern over 15 rounds, controlling for initial climate concern. The model exhibited a very good fit to the data (CFI = 99.2, TLI = 99.2, RMSEA = 0.043 (90% CI = [0.04, 0.046]), SRMR = 0.008). Initial climate concern was strongly and positively associated with the intercept of climate concern (standardized coefficient = 0.92, *SE* = 0.01, *p* < .001), indicating that climate change concern was largely stable across the 15 rounds of the task. Cumulative Pro‐climate box choices had a positive and weak association with the slope of climate concern (standardized *c* = 0.06, *SE* <0.001, *p* = .044). This effect was largely driven by the data from the US sample. When fitting the same model for the US data only, the effect was significant and the effect size was larger (standardized coefficient = 0.50, *SE* = 0.004, *p* = .014), suggesting that in the United States, individual changes in climate concern could be predicted by the number of Pro‐climate box choices. When fitting the same model for the Chinese or German sample separately, the effect of Pro‐climate box choices on the individual slopes of climate concern was insignificant (*p* = .935 and *p* = .176, respectively). This finding corresponds with Figure 5, where visual inspection suggests that individual slopes in climate concern were positive for participants who sampled many Pro‐climate statements and negative for participants with few Pro‐climate box choices.

## DISCUSSION

This cross‐cultural, pre‐registered experiment used a novel sequential information sampling paradigm based on real‐world statements about climate change to investigate information selection, belief updating, and the interplay between the two. Our approach offers insights into how people process information and update their beliefs over time through selecting climate information and disinformation. The sequential nature of the paradigm allowed us to examine aggregated effects of statement sampling over time that may arise from selective exposure to one‐sided messages.

Across all three countries, China, Germany, and the United States, we observed confirmatory box sampling, supporting our first hypotheses. Participants were more likely to choose the box that was better aligned with their initial views on climate change. Exploratively, we found that the preference for the more belief‐congruent box intensified over time, demonstrating that the more participants learned about both boxes, the more selective they became. This finding that confirmatory source selection increased over time is consistent with reinforcement learning and Bayesian belief updating, where a belief‐confirming source can be perceived as increasingly credible (Druckman & McGrath, 2019). One might also become information fatigued, which may create a need for messages that are easier to process, which are more likely to be belief‐congruent. Belief‐congruent messages cause less cognitive dissonance and can be more easily integrated into one's existing belief system and be more emotionally rewarding (Jonas et al., 2001).

Confirmatory information search can be exacerbated by a confirmatory evaluation of climate‐related messages. In our study, participants agreed more with statements that confirmed their initial beliefs and rejected those that did not. Again, there was a temporal dynamic such that the confirmatory evaluation became stronger over time. After participants had formed an impression of a source, similar to a halo effect, this impression shaped how its messages were evaluated. This underscores that messages are not interpreted in a vacuum, but are rather filtered through the lens of the audience's perception of the messenger. The temporal effects were stronger for the Anti‐climate messages, which speaks to their subtlety. Contextualizing these messages as coming from a generally anti‐climate source allowed participants to reject or accept them more strongly depending on their climate beliefs. Comparing confirmatory evaluations between countries, we found that it was stronger in the United States and in Germany than in China. This might indicate that in societies with higher attitudinal and political polarization of climate change, statements are accepted or rejected more decisively than in the absence of a polarized discourse (Hutchens et al., 2019).

Confirming our second hypothesis, climate change concern varied systematically after box choices: it was higher than a participant's mean climate concern after reading a Pro‐climate statement and lower after reading an Anti‐climate statement. We interpret the findings as evidence that the real‐world climate statements exerted persuasive effects among diverse audiences across various national contexts. We found that the statements influenced momentary variations in climate change concern across the entire sample, which is in line with existing findings that both attitude‐congruent and ‐incongruent messages tend to be persuasive (Guess & Coppock, 2020; Haglin, 2017; Levitan & Visser, 2008). The effects of the statements on climate change concern were small but significant and consistent across all countries. Small effect sizes are not surprising given that single statements are unlikely to drastically alter climate change concern. Climate change concern is likely influenced incrementally by numerous messages over prolonged periods and can be reinforced by the algorithmic curation of online media.

The question then becomes when and how these two effects – persuasive messages and confirmatory information search and processing – might turn into a positive feedback loop. This would be indicated if initial opinions become more extreme over time, as preferentially seeking and accepting belief‐confirming statements can continually reinforce existing views (Stroud, 2010). On the population level, this would manifest in a widening gap between people with initially low versus high climate change concern. In the US sample, we observed evidence for an interplay between confirmatory statement selection and cumulative effects on climate concern. While already starting at strongly different levels, climate change concern continued to diverge over the course of the experiment between participants who predominantly chose the Pro‐ or Anti‐climate box (Figure 5). In Germany and in China, the number of Pro‐climate box choices did not significantly predict individual changes in climate change concern, which suggests that the persuasive effects of reading many Pro‐ or Anti‐climate statements did not accumulate and carry over across rounds.

These empirical findings are consistent with predictions from the reinforcing spirals model which posits that in non‐polarized contexts, attitudes tend to remain relatively stable over time (Slater, 2015). In politically polarized societies, however, attitudes can drift further apart through selective exposure to partisan or one‐sided media consumption. It is assumed that in highly polarized environments, partisan identity can evolve into an important social identity and people rely on political leaders of their in‐group when taking positions on political or societal issues (Carmichael & Brulle, 2017; Greene, 2004; Hahnel & Brosch, 2016). Once an issue is associated with partisan identity, besides accuracy motivation, individuals can have identity‐protecting motives and a desire to affirm their group identity by assuming aligned partisan positions (Kahan et al., 2007, 2011). This identity‐protective motivation can strengthen belief‐confirming information search and processing patterns.

Our data can moreover be interpreted in line with theories on cultural cognition and the effect of political polarization on attitude formation. The United States ranks among the most polarized countries with respect to climate‐related issues (Egan & Mullin, 2017; McCright & Dunlap, 2011). A similar trend, with a time lag, is found in Germany where political polarization of climate issues has increased over recent years (Coffé et al., 2025). In our Western samples, this is reflected in moderate to high correlations between climate change beliefs and political orientation and worldviews (see Supporting Information). In China, we could not assess political attitudes, but given the low dispersion in climate change beliefs/concern and the different political system, it is unlikely that political worldviews were strongly related to climate views (Liu, 2023). Surveys suggest that polarization of climate attitudes in China is lower compared with Western countries (Yang et al., 2021). Germany seems to occupy some type of middle ground where results were similar to the U.S. sample, and confirmatory behaviour was stronger compared with China. But in Germany, there was no evidence for reinforcing spirals. This does not imply that reinforcing spirals are non‐existent in Germany; as stated above, our experimental paradigm could only give a brief snapshot into belief formation processes that may fully unfold only over longer time spans.

Surprisingly, while belief‐confirmatory effects were smaller in China and attitudinal polarization was low, Chinese participants responded more strongly to Pro‐ and Anti‐climate statements in their responses on momentary climate concern. That is, momentary climate change concern increased [decreased] more strongly in China after reading a Pro‐ [Anti‐] climate statement than in the Western countries. This may be interpreted in a way that in a less polarized context, Chinese participants were more open and responsive to new arguments. The messages may have momentarily reduced or increased their climate concern, but participants did not integrate this into their overall climate change concern, such that the effects faded immediately. Alternatively, the stronger responsiveness to climate messages may also be a result of greater social desirability or acquiescence.

Lastly, our research can offer some cautious optimism for practical implications to disperse the effects of disinformation. While nearly all participants read multiple disinformation statements, most of them did not exhibit an overall downward trend in climate change concern. This supports the notion that while climate disinformation can negatively affect everyone, specific population segments may be more susceptible to it (González‐Bailón et al., 2023; McCright et al., 2016; Sultan et al., 2024). Our findings suggest that accurate scientific information can have protective effects against the influence of disinformation, which underscores the importance of maximizing encounters with credible climate content. Removing disinformation in the online sphere is notoriously difficult, and the boundaries between free expressions of thoughts and disinformation campaigns can be blurred. Fact‐checking media articles and online posts is resource‐intensive, unscalable, and also contentious (Graves, 2018; Pennycook et al., 2020). As some social media platforms turn away from fact checking and content moderation, alternative strategies are needed (CBS News, 2025; Zannettou, 2021). Interspersing online news feeds with officially verified information might be a promising intervention that merits further investigation. Redirecting people towards trustworthy science communicators that appeal to diverse populations and universally agreed values can be key to reducing the impact of systematic climate disinformation.

## LIMITATIONS

A key consideration regarding the ecological validity and generalizability of our findings is the abstract and stylized nature of our experimental design. Our paradigm is a simplification of real‐world information environments which typically offer a multitude of content, cover versatile topics, and are designed to maximize engagement time. Moreover, aspects such as avoidance of potentially unsettling topics like climate change or the distractive influence of other content likely influence climate information search in the real world (Heseltine et al., 2024). Additionally, algorithmic curation may reinforce existing preferences for either Pro‐ or Anti‐climate content by disproportionately displaying content from one side (Spohr, 2017). Our experimental task, based on a binary design, could not reflect such real‐world influences on information search and processing. However, this controlled design allowed us to isolate the effects of confirmatory information search and processing on attitude formation. Future research should expand on this design to investigate attitude formation in more diverse and naturalistic settings, potentially incorporating a wider array of sources or choice complexities.

A potential limitation of our research is that the Pro‐ and Anti‐climate statements were derived from the platform X, which is not available in China. While the authors of the statements were selected to be diverse and come from 19 different countries, none of the statements was from a Chinese author. Therefore, the narratives reflected in these statements may have been less familiar in the Chinese sample. We carefully checked that the statements did not include specific references to places or people or cultural references or required background knowledge to be understandable. Utilizing a database of diverse and empirically pretested, real‐world social media posts, as conducted here, is an important step to increase ecological validity and reduce the likelihood of cultural biases (Westfall et al., 2014). Unlike artificial stimuli often used in experimental research, real‐world posts better resemble messages encountered in everyday life (Pennycook et al., 2021). Nevertheless, we cannot fully rule out that the statements may have sounded less plausible to a Chinese compared with a Western audience.

Importantly, our paradigm does not afford strict causal claims about the link between information search and attitude changes. Since participants could freely choose between the boxes, the selection effect and changes in climate attitudes might have been caused by a third unobserved variable. Therefore, while our findings are consistent with theoretical models of reinforcing spirals or cumulative exposure, the current design cannot differentiate the causal effect of the choices themselves from other time‐varying factors that may have influenced changes in climate concern. However, given that endogenous sampling tasks offer important advantages over exogenous content presentation, we believe that our results provide circumstantial evidence for the persuasive effects of the climate messages.

## CONCLUSION

Navigating the complex online information environment surrounding climate change requires individuals to discern credible sources from competing and counter‐factual voices. This study contributes to our understanding of the interplay between selective exposure, belief updating, and attitude polarization in the context of climate change communication. In an abstract mini‐simulation of online information search, we found a divergence of attitudes in the United States that is consistent with existing theories of belief formation and reinforcing spiral. We also demonstrate that against diverse cultural backdrops, processes of belief unfold differently, highlighting the role of contextual factors on the emergence of national climate discourses. Providing widespread access to and fostering trust in credible scientific information on climate change is key to countering the influence of climate disinformation. Ultimately, effective science communication should empower individuals to make better informed decisions and can contribute to public discourses with a better understanding of the scientific evidence.

## AUTHOR CONTRIBUTIONS

**Zahra Rahmani Azad:** Conceptualization; software; data curation; formal analysis; writing – original draft; writing – review and editing; methodology; investigation. **Tobia Spampatti:** Conceptualization; methodology; writing – review and editing; supervision. **Sebastian Gluth:** Conceptualization; methodology; writing – review and editing; supervision. **Kim‐Pong Tam:** Conceptualization; methodology; writing – review and editing; supervision. **Ulf J. J. Hahnel:** Conceptualization; methodology; writing – review and editing; supervision; funding acquisition.

## FUNDING INFORMATION

This research was funded as part of the Eccellenza Grant (PCEFPI_203283) from the Swiss National Science Foundation awarded to UJJH. TS thanks the Swiss National Science Foundation for providing individual career funding (SNSF Postdoc.Mobility fellowship P500PS_225577). SG acknowledges support by the European Research Council (ERC) under the European Union's Horizon 2020 Research and Innovation program (grant agreement no. 948545).

## CONFLICT OF INTEREST STATEMENT

The authors have no competing interests to declare.

## Supporting information


Data S1:

## Data Availability

Supporting Information, data and code for all reported analyses are available on OSF (https://osf.io/w4kdh).

## Footnotes

^1^
We will also use these terms in the manuscript while realizing that the believer/sceptic dichotomy is a simplified perspective on a wide range of different positions on climate change.

## References

[bjop70028-bib-0001] Amasino, D. R. , Pace, D. D. , & van der Weele, J. J. (2024). Fair shares and selective attention. American Economic Journal: Microeconomics, 16(4), 259–290. 10.1257/mic.20220275

[bjop70028-bib-0002] Ballew, M. T. , Thomas‐Walters, L. , Goldberg, M. H. , Verner, M. , Lu, J. , Marshall, J. , Rosenthal, S. A. , & Leiserowitz, A. (2025). Climate change messages can promote support for climate action globally. Global Environmental Change, 90, 102951. 10.1016/j.gloenvcha.2024.102951

[bjop70028-bib-0003] Bates, D. , Mächler, M. , Bolker, B. , & Walker, S. (2015). Fitting linear mixed‐effects models using lme4. Journal of Statistical Software, 67(1), 1–48. 10.18637/jss.v067.i01

[bjop70028-bib-0004] Berkebile‐Weinberg, M. , Goldwert, D. , Doell, K. C. , Van Bavel, J. J. , & Vlasceanu, M. (2024). The differential impact of climate interventions along the political divide in 60 countries. Nature Communications, 15(1), 3885. 10.1038/s41467-024-48112-8 PMC11078920 38719845

[bjop70028-bib-0005] Bolin, J. L. , & Hamilton, L. C. (2018). The news you choose: News media preferences amplify views on climate change. Environmental Politics, 27, 455–476. 10.1080/09644016.2018.1423909

[bjop70028-bib-0006] Bolsen, T. , & Shapiro, M. A. (2018). The US news media, polarization on climate change, and pathways to effective communication. Environmental Communication, 12, 149–163. 10.1080/17524032.2017.1397039

[bjop70028-bib-0007] Brüggemann, M. , & Engesser, S. (2017). Beyond false balance: How interpretive journalism shapes media coverage of climate change. Global Environmental Change, 42, 58–67. 10.1016/j.gloenvcha.2016.11.004

[bjop70028-bib-0008] Brulle, R. J. (2018). The climate lobby: A sectoral analysis of lobbying spending on climate change in the USA, 2000 to 2016. Climatic Change, 149(3), 289–303. 10.1007/s10584-018-2241-z

[bjop70028-bib-0009] Brulle, R. J. , Aronczyk, M. , & Carmichael, J. (2020). Corporate promotion and climate change: An analysis of key variables affecting advertising spending by major oil corporations, 1986–2015. Climatic Change, 159(1), 87–101. 10.1007/s10584-019-02582-8

[bjop70028-bib-0010] Brulle, R. J. , & Downie, C. (2022). Following the money: Trade associations, political activity and climate change. Climatic Change, 175(3), 11. 10.1007/s10584-022-03466-0

[bjop70028-bib-0011] Carmichael, J. T. , & Brulle, R. J. (2017). Elite cues, media coverage, and public concern: An integrated path analysis of public opinion on climate change, 2001–2013. Environmental Politics, 26, 232–252. 10.1080/09644016.2016.1263433

[bjop70028-bib-0012] Carmichael, J. T. , Brulle, R. J. , & Huxster, J. K. (2017). The great divide: Understanding the role of media and other drivers of the partisan divide in public concern over climate change in the USA, 2001–2014. Climatic Change, 141(4), 599–612. 10.1007/s10584-017-1908-1

[bjop70028-bib-0013] CBS News . (2025). Meta to end fact‐checking, replacing it with community‐driven system akin to Elon Musk's X . https://www.cbsnews.com/news/meta‐facebook‐instagram‐fact‐checking‐mark‐zuckerberg/

[bjop70028-bib-0014] Chopra, F. , Haaland, I. , & Roth, C. (2024). The demand for news: Accuracy concerns versus belief confirmation motives. The Economic Journal, 134(661), 1806–1834. 10.1093/ej/ueae019

[bjop70028-bib-0015] Chu, J. , Zhu, Y. , & Ji, J. (2023). Characterizing the semantic features of climate change misinformation on Chinese social media. Public Understanding of Science, 32(7), 845–859. 10.1177/09636625231166542 37162274

[bjop70028-bib-0016] Coan, T. G. , Boussalis, C. , Cook, J. , & Nanko, M. O. (2021). Computer‐assisted classification of contrarian claims about climate change. Scientific Reports, 11(1), 22320. 10.1038/s41598-021-01714-4 34785707 PMC8595491

[bjop70028-bib-0017] Coffé, H. , Crawley, S. , & Givens, J. (2025). Growing polarisation: Ideology and attitudes towards climate change. West European Politics, 1–29, 1–29. 10.1080/01402382.2024.2435727

[bjop70028-bib-0018] Cook, J. , Oreskes, N. , Doran, P. T. , Anderegg, W. R. L. , Verheggen, B. , Maibach, E. W. , Carlton, J. S. , Lewandowsky, S. , Skuce, A. G. , Green, S. A. , Nuccitelli, D. , Jacobs, P. , Richardson, M. , Winkler, B. , Painting, R. , & Rice, K. (2016). Consensus on consensus: A synthesis of consensus estimates on human‐caused global warming. Environmental Research Letters, 11(4), 048002. 10.1088/1748-9326/11/4/048002

[bjop70028-bib-0019] Cook, J. , Supran, G. , Lewandowsky, S. , Oreskes, N. , & Maibach, E. (2019). America Misled: How the fossil fuel industry deliberately misled Americans about climate change . https://policycommons.net/artifacts/2193396/america‐misled/2949373/

[bjop70028-bib-0020] Corner, A. , Whitmarsh, L. , & Xenias, D. (2012). Uncertainty, scepticism and attitudes towards climate change: Biased assimilation and attitude polarisation. Climatic Change, 114(3–4), 463–478. 10.1007/s10584-012-0424-6

[bjop70028-bib-0021] Doyle, J. (2011). Where has all the oil gone? BP branding and the discursive elimination of climate change risk. In N. Heffernan & D. A. Wragg (Eds.), Culture, environment and ecopolitics (pp. 200–225). Cambridge Scholars Publishing.

[bjop70028-bib-0022] Druckman, J. N. , & McGrath, M. C. (2019). The evidence for motivated reasoning in climate change preference formation. Nature Climate Change, 9(2), 111–119. 10.1038/s41558-018-0360-1

[bjop70028-bib-0023] Du, J. , Liu, J. C.‐E. , & Mix, T. L. (2024). China deserves its hamburger: The controversy over WildAid's Shu Shi campaign in China. Environmental Sociology, 10(3), 321–332. 10.1080/23251042.2024.2319370

[bjop70028-bib-0024] Dunlap, R. E. , & McCright, A. M. (2010). Climate change denial: Sources, actors and strategies. In Routledge handbook of climate change and society. Routledge.

[bjop70028-bib-0025] Dunlap, R. E. , McCright, A. M. , & Yarosh, J. H. (2016). The political divide on climate change: Partisan polarization widens in the U.S. Environment: Science and Policy for Sustainable Development, 58(5), 4–23. 10.1080/00139157.2016.1208995

[bjop70028-bib-0026] Egan, P. J. , & Mullin, M. (2017). Climate change: US public opinion. Annual Review of Political Science, 20(1), 209–227. 10.1146/annurev-polisci-051215-022857

[bjop70028-bib-0027] Falkenberg, M. , Galeazzi, A. , Torricelli, M. , Di Marco, N. , Larosa, F. , Sas, M. , Mekacher, A. , Pearce, W. , Zollo, F. , Quattrociocchi, W. , & Baronchelli, A. (2022). Growing polarization around climate change on social media. Nature Climate Change, 12(12), 1114–1121. 10.1038/s41558-022-01527-x

[bjop70028-bib-0028] Farrell, J. (2016). Corporate funding and ideological polarization about climate change. Proceedings of the National Academy of Sciences, 113(1), 92–97. 10.1073/pnas.1509433112 PMC4711825 26598653

[bjop70028-bib-0029] Fielding, K. S. , & Hornsey, M. J. (2016). A social identity analysis of climate change and environmental attitudes and behaviors: Insights and opportunities. Frontiers in Psychology, 7, 121. 10.3389/fpsyg.2016.00121 26903924 PMC4749709

[bjop70028-bib-0030] Fischer, O. , & Frey, R. (2023). The many flavors of polarization: A systematic comparison of different conceptualizations and contexts. OSF. 10.31234/osf.io/bv496

[bjop70028-bib-0031] Freelon, D. , & Wells, C. (2020). Disinformation as political communication. Political Communication, 37(2), 145–156. 10.1080/10584609.2020.1723755

[bjop70028-bib-0032] Freeman, J. B. , & Dale, R. (2013). Assessing bimodality to detect the presence of a dual cognitive process. Behavior Research Methods, 45(1), 83–97. 10.3758/s13428-012-0225-x 22806703

[bjop70028-bib-0033] Garrett, R. K. (2009). Echo chambers online?: Politically motivated selective exposure among internet news users. Journal of Computer‐Mediated Communication, 14(2), 265–285. 10.1111/j.1083-6101.2009.01440.x

[bjop70028-bib-0034] Gluth, S. , Deakin, J. , & Rieskamp, J. (2024). A Theory of Multi‐Attribute Search and Choice. OSF. 10.31234/osf.io/3qzak

[bjop70028-bib-0035] Goldberg, M. H. , Gustafson, A. , van der Linden, S. , Rosenthal, S. A. , & Leiserowitz, A. (2022). Communicating the scientific consensus on climate change: Diverse audiences and effects over time. Environment and Behavior, 54(7–8), 1133–1165. 10.1177/00139165221129539

[bjop70028-bib-0036] González‐Bailón, S. , Lazer, D. , Barberá, P. , Zhang, M. , Allcott, H. , Brown, T. , Crespo‐Tenorio, A. , Freelon, D. , Gentzkow, M. , Guess, A. M. , Iyengar, S. , Kim, Y. M. , Malhotra, N. , Moehler, D. , Nyhan, B. , Pan, J. , Rivera, C. V. , Settle, J. , Thorson, E. , … Tucker, J. A. (2023). Asymmetric ideological segregation in exposure to political news on Facebook. Science, 381(6656), 392–398. 10.1126/science.ade7138 37499003

[bjop70028-bib-0037] Goodrich, B. , Fenton, M. , Penn, J. , Bovay, J. , & Mountain, T. (2023). Battling bots: Experiences and strategies to mitigate fraudulent responses in online surveys. Applied Economic Perspectives and Policy, 45(2), 762–784. 10.1002/aepp.13353

[bjop70028-bib-0038] Graaf, J. D. , Bal, M. , de Wit, J. , & Stok, M. (2024). Climate change doom communication from a fear appeal perspective. European Journal of Health Communication, 5(4), 26–50. 10.47368/ejhc.2024.402

[bjop70028-bib-0039] Graves, L. (2018). Boundaries not drawn: Mapping the institutional roots of the global fact‐checking movement. Journalism Studies, 19(5), 613–631. 10.1080/1461670X.2016.1196602

[bjop70028-bib-0040] Greene, S. (2004). Social identity theory and party identification. Social Science Quarterly, 85(1), 136–153. 10.1111/j.0038-4941.2004.08501010.x

[bjop70028-bib-0041] Guenther, L. , Brüggemann, M. , & Elkobros, S. (2022). From global doom to sustainable solutions: International news magazines' multimodal framing of our future with climate change. Journalism Studies, 23(1), 131–148. 10.1080/1461670X.2021.2007162

[bjop70028-bib-0042] Guess, A. , & Coppock, A. (2020). Does counter‐attitudinal information cause backlash? Results from three large survey experiments. British Journal of Political Science, 50(4), 1497–1515. 10.1017/S0007123418000327

[bjop70028-bib-0043] Guo, J. , Huang, X. , & Fang, K. (2023). Authoritarian environmentalism as reflected in the journalistic sourcing of climate change reporting in China. Environmental Communication, 17(5), 502–517. 10.1080/17524032.2023.2223774

[bjop70028-bib-0044] Haglin, K. (2017). The limitations of the backfire effect. Research & Politics, 4(3), 2053168017716547. 10.1177/2053168017716547

[bjop70028-bib-0045] Hahnel, U. J. J. , & Brosch, T. (2016). Seeing Green: A perceptual model of identity‐based climate change judgments. Psychological Inquiry, 27(4), 310–318. 10.1080/1047840X.2016.1215205

[bjop70028-bib-0046] Heseltine, M. , Barnehl, H. , & Wojcieszak, M. (2024). Partisan temporal selective news avoidance: Evidence from online trace data. American Journal of Political Science, 0, 1–18. 10.1111/ajps.12944

[bjop70028-bib-0047] Hornsey, M. J. (2021). The role of worldviews in shaping how people appraise climate change. Current Opinion in Behavioral Sciences, 42, 36–41. 10.1016/j.cobeha.2021.02.021

[bjop70028-bib-0048] Hornsey, M. J. , Harris, E. A. , & Fielding, K. S. (2018). Relationships among conspiratorial beliefs, conservatism and climate scepticism across nations. Nature Climate Change, 8(7), 614–620. 10.1038/s41558-018-0157-2

[bjop70028-bib-0049] Hutchens, M. J. , Hmielowski, J. D. , & Beam, M. A. (2019). Reinforcing spirals of political discussion and affective polarization. Communication Monographs, 86(3), 357–376. 10.1080/03637751.2019.1575255

[bjop70028-bib-0050] IPCC . (2023). Summary for Policymakers. In Core Writing Team , H. Lee , & J. Romero (Eds.), Climate Change 2023: Synthesis Report. Contribution of Working Groups I, II and III to the Sixth Assessment Report of the Intergovernmental Panel on Climate Change (pp. 1–34). IPCC. 10.59327/IPCC/AR6-9789291691647.001

[bjop70028-bib-0051] Iyengar, S. , & Hahn, K. S. (2009). Red media, blue media: Evidence of ideological selectivity in media use. Journal of Communication, 59(1), 19–39. 10.1111/j.1460-2466.2008.01402.x

[bjop70028-bib-0052] Jonas, E. , Schulz‐Hardt, S. , Frey, D. , & Thelen, N. (2001). Confirmation bias in sequential information search after preliminary decisions: An expansion of dissonance theoretical research on selective exposure to information. Journal of Personality and Social Psychology, 80, 557–571. 10.1037/0022-3514.80.4.557 11316221

[bjop70028-bib-0053] Judge, M. , Kashima, Y. , Steg, L. , & Dietz, T. (2023). Environmental decision‐making in times of polarization. Annual Review of Environment and Resources, 48, 477–503. 10.1146/annurev-environ-112321-115339

[bjop70028-bib-0054] Kahan, D. M. , Braman, D. , Gastil, J. , Slovic, P. , & Mertz, C. K. (2007). Culture and identity‐protective cognition: Explaining the White‐male effect in risk perception. Journal of Empirical Legal Studies, 4(3), 465–505. 10.1111/j.1740-1461.2007.00097.x

[bjop70028-bib-0055] Kahan, D. M. , Jenkins‐Smith, H. , & Braman, D. (2011). Cultural cognition of scientific consensus. Journal of Risk Research, 14(2), 147–174. 10.1080/13669877.2010.511246

[bjop70028-bib-0056] Kerschreiter, R. , Schulz‐Hardt, S. , Mojzisch, A. , & Frey, D. (2008). Biased information search in homogeneous groups: Confidence as a moderator for the effect of anticipated task requirements. Personality and Social Psychology Bulletin, 34(5), 679–691. 10.1177/0146167207313934 18310314

[bjop70028-bib-0057] Knobloch‐Westerwick, S. , & Meng, J. (2009). Looking the other way: Selective exposure to attitude‐consistent and Counterattitudinal political information. Communication Research, 36(3), 426–448. 10.1177/0093650209333030

[bjop70028-bib-0058] Kuznetsova, A. , Brockhoff, P. B. , & Christensen, R. H. B. (2017). lmerTest package: Tests in linear mixed effects models. Journal of Statistical Software, 82(13), 1–26. 10.18637/jss.v082.i13

[bjop70028-bib-0059] Lamb, W. F. , Mattioli, G. , Levi, S. , Roberts, J. T. , Capstick, S. , Creutzig, F. , Minx, J. C. , Müller‐Hansen, F. , Culhane, T. , & Steinberger, J. K. (2020). Discourses of climate delay. Global Sustainability, 3, e17. 10.1017/sus.2020.13

[bjop70028-bib-0060] Levitan, L. C. , & Visser, P. S. (2008). The impact of the social context on resistance to persuasion: Effortful versus effortless responses to counter‐attitudinal information. Journal of Experimental Social Psychology, 44(3), 640–649. 10.1016/j.jesp.2007.03.004

[bjop70028-bib-0061] Lewandowsky, S. (2021). Climate change disinformation and how to combat it. Annual Review of Public Health, 42, 1–21. 10.1146/annurev-publhealth-090419-102409 33355475

[bjop70028-bib-0062] Lewandowsky, S. , Ecker, U. K. H. , Seifert, C. M. , Schwarz, N. , & Cook, J. (2012). Misinformation and its correction: Continued influence and successful Debiasing. Psychological Science in the Public Interest, 13(3), 106–131. 10.1177/1529100612451018 26173286

[bjop70028-bib-0063] Lewandowsky, S. , & van der Linden, S. (2021). Countering misinformation and fake news through inoculation and Prebunking. European Review of Social Psychology, 32(2), 348–384. 10.1080/10463283.2021.1876983

[bjop70028-bib-0064] Lewis, G. B. , Palm, R. , & Feng, B. (2019). Cross‐national variation in determinants of climate change concern. Environmental Politics, 28(5), 793–821. 10.1080/09644016.2018.1512261

[bjop70028-bib-0065] Liu, J. C.‐E. (2015). Low carbon plot: Climate change skepticism with Chinese characteristics. Environmental Sociology, 1(4), 280–292. 10.1080/23251042.2015.1049811

[bjop70028-bib-0066] Liu, J. C.‐E. (2023). Public opinion on climate change in China—Evidence from two national surveys. PLOS Climate, 2(2), e0000065.

[bjop70028-bib-0067] Lynas, M. , Houlton, B. Z. , & Perry, S. (2021). Greater than 99% consensus on human caused climate change in the peer‐reviewed scientific literature. Environmental Research Letters, 16(11), 114005. 10.1088/1748-9326/ac2966

[bjop70028-bib-0068] McCright, A. M. , Charters, M. , Dentzman, K. , & Dietz, T. (2016). Examining the effectiveness of climate change frames in the face of a climate change denial counter‐frame. Topics in Cognitive Science, 8(1), 76–97. 10.1111/tops.12171 26621098

[bjop70028-bib-0069] McCright, A. M. , & Dunlap, R. E. (2011). The politicization of climate change and polarization in the American Public's views of global warming, 2001–2010. The Sociological Quarterly, 52(2), 155–194. 10.1111/j.1533-8525.2011.01198.x

[bjop70028-bib-0070] Meng, K. C. , & Rode, A. (2019). The social cost of lobbying over climate policy. Nature Climate Change, 9(6), 472–476. 10.1038/s41558-019-0489-6

[bjop70028-bib-0071] Newman, T. P. , Nisbet, E. C. , & Nisbet, M. C. (2018). Climate change, cultural cognition, and media effects: Worldviews drive news selectivity, biased processing, and polarized attitudes. Public Understanding of Science, 27(8), 985–1002. 10.1177/0963662518801170 30253695

[bjop70028-bib-0072] Oreskes, N. , & Conway, E. M. (2011). Merchants of doubt: How a handful of scientists obscured the truth on issues from tobacco smoke to global warming. Bloomsbury Publishing.

[bjop70028-bib-0073] Painter, J. , Ettinger, J. , Holmes, D. , Loy, L. , Pinto, J. , Richardson, L. , Thomas‐Walters, L. , Vowles, K. , & Wetts, R. (2023). Climate delay discourses present in global mainstream television coverage of the IPCC's 2021 report. Communications Earth & Environment, 4(1), 118. 10.1038/s43247-023-00760-2

[bjop70028-bib-0074] Pennycook, G. , Bear, A. , Collins, E. T. , & Rand, D. G. (2020). The implied truth effect: Attaching warnings to a subset of fake news headlines increases perceived accuracy of headlines without warnings. Management Science, 66(11), 4944–4957. 10.1287/mnsc.2019.3478

[bjop70028-bib-0075] Pennycook, G. , Binnendyk, J. , Newton, C. , & Rand, D. G. (2021). A practical guide to doing behavioral research on fake news and misinformation. Collabra: Psychology, 7(1), 25293. 10.1525/collabra.25293

[bjop70028-bib-0076] Pennycook, G. , & Rand, D. G. (2021). The psychology of fake news. Trends in Cognitive Sciences, 25(5), 388–402. 10.1016/j.tics.2021.02.007 33736957

[bjop70028-bib-0077] Petersen, A. M. , Vincent, E. M. , & Westerling, A. L. (2019). Discrepancy in scientific authority and media visibility of climate change scientists and contrarians. Nature Communications, 10(1), 3502. 10.1038/s41467-019-09959-4 PMC6692310 31409789

[bjop70028-bib-0078] Pew Research Center . (2022). Climate Change Remains Top Global Threat Across 19‐Country Survey. https://www.pewresearch.org/global/2022/08/31/climate‐change‐remains‐top‐global‐threat‐across‐19‐country‐survey/

[bjop70028-bib-0079] Pringle, A. , & Robbins, D. (2022). From denial to delay: Climate change discourses in Ireland. Administration, 70(3), 59–84. 10.2478/admin-2022-0019

[bjop70028-bib-0080] R Core Team . (2020). R: A language and environment for statistical computing. R Foundation for Statistical Computing. https://www.R‐project.org/

[bjop70028-bib-0081] Roper, J. , Ganesh, S. , & Zorn, T. E. (2016). Doubt, delay, and discourse: Skeptics' strategies to politicize climate change. Science Communication, 38(6), 776–799. 10.1177/1075547016677043

[bjop70028-bib-0082] Rosseel, Y. (2012). Lavaan: An R package for structural equation modeling. Journal of Statistical Software, 48, 1–36. 10.18637/jss.v048.i02

[bjop70028-bib-0083] Slater, M. D. (2007). Reinforcing spirals: The mutual influence of media selectivity and media effects and their impact on individual behavior and social identity. Communication Theory, 17(3), 281–303. 10.1111/j.1468-2885.2007.00296.x

[bjop70028-bib-0084] Slater, M. D. (2015). Reinforcing spirals model: Conceptualizing the relationship between media content exposure and the development and maintenance of attitudes. Media Psychology, 18(3), 370–395. 10.1080/15213269.2014.897236 26366124 PMC4565521

[bjop70028-bib-0085] Spampatti, T. , Brosch, T. , Mumenthaler, C. , & Hahnel, U. J. J. (2025). Blueprint of a smokescreen: Introducing the validated climate disinformation corpus for behavioural research on combating climate disinformation. British Journal of Psychology, 1–25. 10.1111/bjop.70012 PMC13543337 40682248

[bjop70028-bib-0086] Spampatti, T. , Hahnel, U. J. J. , Trutnevyte, E. , & Brosch, T. (2024). Psychological inoculation strategies to fight climate disinformation across 12 countries. Nature Human Behaviour, 8(2), 380–398. 10.1038/s41562-023-01736-0 PMC10896732 38036655

[bjop70028-bib-0087] Spohr, D. (2017). Fake news and ideological polarization: Filter bubbles and selective exposure on social media. Business Information Review, 34(3), 150–160. 10.1177/0266382117722446

[bjop70028-bib-0088] Storozuk, A. , Ashley, M. , Delage, V. , & Maloney, E. A. (2020). Got bots? Practical recommendations to protect online survey data from bot attacks. The Quantitative Methods for Psychology, 16(5), 472–481. 10.20982/tqmp.16.5.p472

[bjop70028-bib-0089] Stroud, N. J. (2010). Polarization and partisan selective exposure. Journal of Communication, 60(3), 556–576. 10.1111/j.1460-2466.2010.01497.x

[bjop70028-bib-0090] Sultan, M. , Tump, A. N. , Ehmann, N. , Lorenz‐Spreen, P. , Hertwig, R. , Gollwitzer, A. , & Kurvers, R. H. J. M. (2024). Susceptibility to online misinformation: A systematic meta‐analysis of demographic and psychological factors. Proceedings of the National Academy of Sciences, 121(47), e2409329121. 10.1073/pnas.2409329121 PMC11588074 39531500

[bjop70028-bib-0091] Sunstein, C. R. , Bobadilla‐Suarez, S. , Lazzaro, S. C. , & Sharot, T. (2016). How people update beliefs about climate change: Good news and bad news. Cornell Law Review, 102, 1431.

[bjop70028-bib-0092] Supran, G. , & Oreskes, N. (2017). Assessing ExxonMobil's climate change communications (1977–2014). Environmental Research Letters, 12(8), 084019. 10.1088/1748-9326/aa815f

[bjop70028-bib-0093] Supran, G. , & Oreskes, N. (2021). Rhetoric and frame analysis of ExxonMobil's climate change communications. One Earth, 4(5), 696–719. 10.1016/j.oneear.2021.04.014

[bjop70028-bib-0094] Supran, G. , Rahmstorf, S. , & Oreskes, N. (2023). Assessing ExxonMobil's global warming projections. Science, 379(6628), eabk0063. 10.1126/science.abk0063 36634176

[bjop70028-bib-0095] Talluri, B. C. , Urai, A. E. , Tsetsos, K. , Usher, M. , & Donner, T. H. (2018). Confirmation bias through selective overweighting of choice‐consistent evidence. Current Biology, 28(19), 3128–3135. 10.1016/j.cub.2018.07.052 30220502

[bjop70028-bib-0096] Tam, K.‐P. , & Chan, H.‐W. (2023). Conspiracy theories and climate change: A systematic review. Journal of Environmental Psychology, 91, 102129. 10.1016/j.jenvp.2023.102129

[bjop70028-bib-0097] Tam, K.‐P. , Leung, A.K.‐y. , & Clayton, S. (2021). Research on climate change in social psychology publications: A systematic review. Asian Journal of Social Psychology, 24(2), 117–143. 10.1111/ajsp.12477

[bjop70028-bib-0098] van Valkengoed, A. M. , Steg, L. , & Perlaviciute, G. (2021). Development and validation of a climate change perceptions scale. Journal of Environmental Psychology, 76, 101652. 10.1016/j.jenvp.2021.101652

[bjop70028-bib-0099] Većkalov, B. , Geiger, S. J. , Bartoš, F. , White, M. P. , Rutjens, B. T. , van Harreveld, F. , Stablum, F. , Akın, B. , Aldoh, A. , Bai, J. , Berglund, F. , Bratina Zimic, A. , Broyles, M. , Catania, A. , Chen, A. , Chorzępa, M. , Farahat, E. , Götz, J. , Hoter‐Ishay, B. , … van der Linden, S. (2024). A 27‐country test of communicating the scientific consensus on climate change. Nature Human Behaviour, 8(10), 1892–1905. 10.1038/s41562-024-01928-2 PMC11493676 39187712

[bjop70028-bib-0100] Vlasceanu, M. , Doell, K. C. , Bak‐Coleman, J. B. , Todorova, B. , Berkebile‐Weinberg, M. M. , Grayson, S. J. , Patel, Y. , Goldwert, D. , Pei, Y. , Chakroff, A. , Pronizius, E. , van den Broek, K. L. , Vlasceanu, D. , Constantino, S. , Morais, M. J. , Schumann, P. , Rathje, S. , Fang, K. , Aglioti, S. M. , … Van Bavel, J. J. (2024). Addressing climate change with behavioral science: A global intervention tournament in 63 countries. Science Advances, 10(6), eadj5778. 10.1126/sciadv.adj5778 38324680 PMC10849597

[bjop70028-bib-0101] Wang, B. , & Zhou, Q. (2020). Climate change in the Chinese mind: An overview of public perceptions at macro and micro levels. Wiley Interdisciplinary Reviews: Climate Change, 11(3), e639. 10.1002/wcc.639

[bjop70028-bib-0102] West, E. A. , & Iyengar, S. (2022). Partisanship as a social identity: Implications for polarization. Political Behavior, 44(2), 807–838. 10.1007/s11109-020-09637-y

[bjop70028-bib-0103] Westfall, J. , Kenny, D. A. , & Judd, C. M. (2014). Statistical power and optimal design in experiments in which samples of participants respond to samples of stimuli. Journal of Experimental Psychology: General, 143(5), 2020–2045. 10.1037/xge0000014 25111580

[bjop70028-bib-0104] Wickham, H. , Averick, M. , Bryan, J. , Chang, W. , McGowan, L. D. , François, R. , Grolemund, G. , Hayes, A. , Henry, L. , Hester, J. , Kuhn, M. , Pedersen, T. L. , Miller, E. , Bache, S. M. , Müller, K. , Ooms, J. , Robinson, D. , Seidel, D. P. , Spinu, V. , … Yutani, H. (2019). Welcome to the tidyverse. Journal of Open Source Software, 4(43), 1686. 10.21105/joss.01686

[bjop70028-bib-0105] Winter, K. , Hornsey, M. J. , Pummerer, L. , & Sassenberg, K. (2024). Public agreement with misinformation about wind farms. Nature Communications, 15(1), 8888. 10.1038/s41467-024-53278-2 PMC11480317 39406698

[bjop70028-bib-0106] Wojcieszak, M. , & Garrett, R. K. (2018). Social identity, selective exposure, and affective polarization: How priming National Identity Shapes Attitudes toward Immigrants via News Selection. Human Communication Research, 44(3), 247–273. 10.1093/hcr/hqx010

[bjop70028-bib-0107] Yang, J. , Gounaridis, D. , Liu, M. , Bi, J. , & Newell, J. P. (2021). Perceptions of climate change in China: Evidence from surveys of residents in six cities. Earth's Future, 9(12), e2021EF002144. 10.1029/2021EF002144

[bjop70028-bib-0108] Zannettou, S. (2021). “I won the election!”: An empirical analysis of soft moderation interventions on twitter. Proceedings of the International AAAI Conference on Web and Social Media, 15, 865–876. 10.1609/icwsm.v15i1.18110

[bjop70028-bib-0109] Zeng, L. (2022). Chinese public perception of climate change on social media: An investigation based on data mining and text analysis. Journal of Environmental and Public Health, 2022(1), 6294436. 10.1155/2022/6294436 36060878 PMC9433270

