# Supplementary material for: Sampling and processing of climate change information and disinformation across three diverse countries
Source: Br J Psychol. 2025 Sep 25;117(Suppl 1):S90–S112. doi: 10.1111/bjop.70028 (PMC13543321; doi:10.1111/bjop.70028)
Supplement: Supplementary file 1 [file BJOP-117-S90-s001.pdf]

Supplementary Information for Sampling and processing of climate change information and disinformation across three culturally diverse countries

Zahra Rahmani Azad[1], Tobia Spampatti[2,3,4], Sebastian Gluth[5], Kim-Pong Tam[6], Ulf J.J. Hahnel[1,7]

[1] Faculty of Psychology, University of Basel
[2] Department of Psychology University of Geneva, Switzerland
[3] Swiss Center for Affective Sciences, University of Geneva, Switzerland
[4] Center for Conflict and Cooperation, New York University, United States of America
[5] Department of Psychology, University of Hamburg, Germany
[6]The Hong Kong University of Science and Technology, Hong Kong
[7]Institute for Sustainability Psychology, Leuphana University Lüneburg, Germany

Data and Analysis Scripts can be found here: https://osf.io/w4kdh/

Author note

Correspondence concerning this article should be addressed to Zahra Rahmani Azad, Department Psychology, University of Basel, Missionsstrasse 62A, 4056 Basel, Switzerland. E-mail: zahra.rahmani@unibas.ch

Supplementary Information for Sampling and processing of climate change information and disinformation across three culturally diverse countries

Demographic Composition and Representativeness

Age Group	China Count	China Actual	China Planned	Germany Count	Germany Actual	Germany Planned	U.S. Count	U.S. Actual	U.S. Planned
<29	178	23.5%	23.4%	107	13.5%	15.5%	124	18.4%	22.4%
30s	223	29.5%	19.5%	168	21.1%	18.5%	140	20.8%	19%
40s	168	22.2%	19.5%	158	19.9%	17%	126	18.7%	17.2%
50s	110	14.5%	19.5%	156	19.6%	23.5%	108	16%	17.4%
>60	78	10.3%	18.1%	206	25.9%	25.5%	176	26.1%	24%

Table S1. Participants by age categories compared to planned distribution for representativeness.

Sex	China Count	China Actual	China Planned	Germany Count	Germany Actual	Germany Planned	U.S. Count	U.S. Actual	U.S. Planned
female	396	52.3%	48.1%	422	53.1%	51%	371	55%	50.5%
male	361	47.7%	51.9%	373	46.9%	49%	303	45%	49.5%

Table S2. Participant by sex compared to planned distribution for representativeness.

Residential Area	China Count	China Actual	Germany Count	Germany Actual	Germany Planned	U.S. Count	U.S. Actual	U.S. Planned
rural	27	3.6	201	25.3%	18	116	17.2%	14
suburban	181	23.9	311	39.1%	47	349	51.8%	55
urban	549	72.5	283	35.6%	35	209	31%	31

Table S3. Participant by residential area compared to planned distribution for representativeness. There were no quotas for China.

Income	Germany Count	Germany Actual	Germany Planned	U.S. Count	U.S. Actual	U.S. Planned
< 1600€ <$28k	125	15.7%	20%	149	22.1%	20%
1600 - 2300€ $28k - 55k	156	19.6%	20%	162	24%	20%
2301 - 3300€ $55k - 81k	197	24.8%	20%	169	25.1%	20%
3301 - 4700€ $81k - 149k	149	18.7%	20%	128	19%	20%
> 4700€ >$150k	168	21.1%	20%	66	9.8%	20%

Table S4. Participant by income quantiles for U.S. and Germany compared to planned distribution for representativeness. Monthly net household income in Germany. Yearly gross household income in the U.S. For China, there were no income quotas, and income is very heterogeneous across regions, therefore no income groups could be applied.

Material and Self-Report Scales

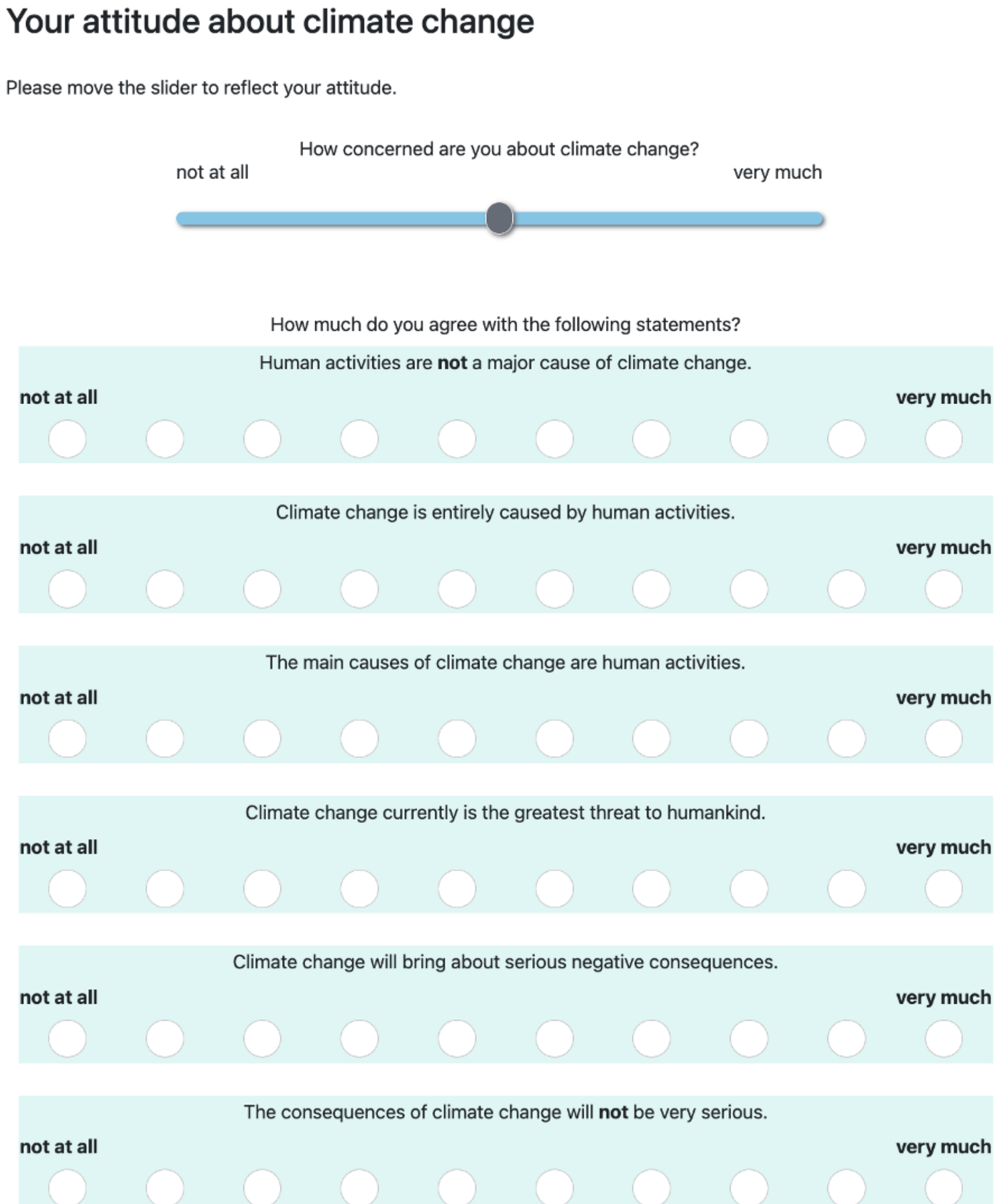

Your attitude about climate change

Please move the slider to reflect your attitude.

How concerned are you about climate change?

not at all — very much

How much do you agree with the following statements?

Human activities are **not** a major cause of climate change.

not at all — **very much**

Climate change is entirely caused by human activities.

not at all — **very much**

The main causes of climate change are human activities.

not at all — **very much**

Climate change currently is the greatest threat to humankind.

not at all — **very much**

Climate change will bring about serious negative consequences.

not at all — **very much**

The consequences of climate change will **not** be very serious.

not at all — **very much**

Figure S1. Screenshot showing items used to assess initial climate beliefs (van Valkengoed et al., 2021) and slider for initial climate change concern.

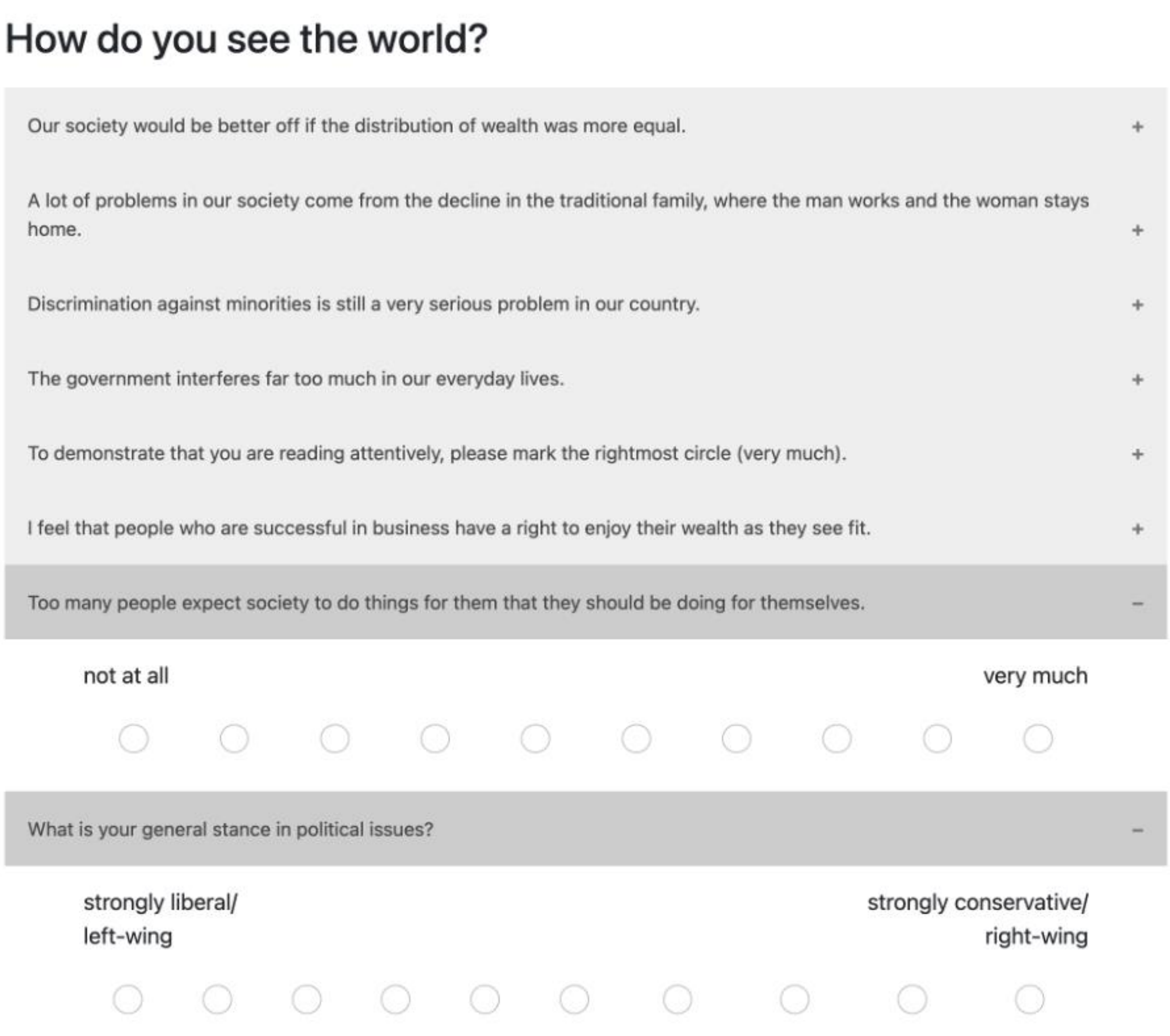

How do you see the world?

Our society would be better off if the distribution of wealth was more equal. +

A lot of problems in our society come from the decline in the traditional family, where the man works and the woman stays home. +

Discrimination against minorities is still a very serious problem in our country. +

The government interferes far too much in our everyday lives. +

To demonstrate that you are reading attentively, please mark the rightmost circle (very much). +

I feel that people who are successful in business have a right to enjoy their wealth as they see fit. +

Too many people expect society to do things for them that they should be doing for themselves. –

not at all … very much

What is your general stance in political issues? –

strongly liberal/ left-wing … strongly conservative/ right-wing

Figure S2. Screenshot showing items used to assess Worldviews and Political Attitudes (van Valkengoed et al., 2021). These questions were not assessed in China. These items (three each for Egalitarian vs. Hierarchicalist and Collectivist vs. Individualist views) were a subset of items by Kahan et al. (2007). This short 6-item version has previously been used in other studies (Truelove & Greenberg, 2013).

As described in the Method section of the Manuscript (see also Figure 1 in the manuscript), we asked participants about their impression of the boxes during the sampling tasks. These questions were asked after round 5, round 10 and round 15. Note that after round 15, there were two additional questions per box ("*What do you think is the political orientation of Box _?* " and "*How much did the statements from Box_ change your opinion?* ").

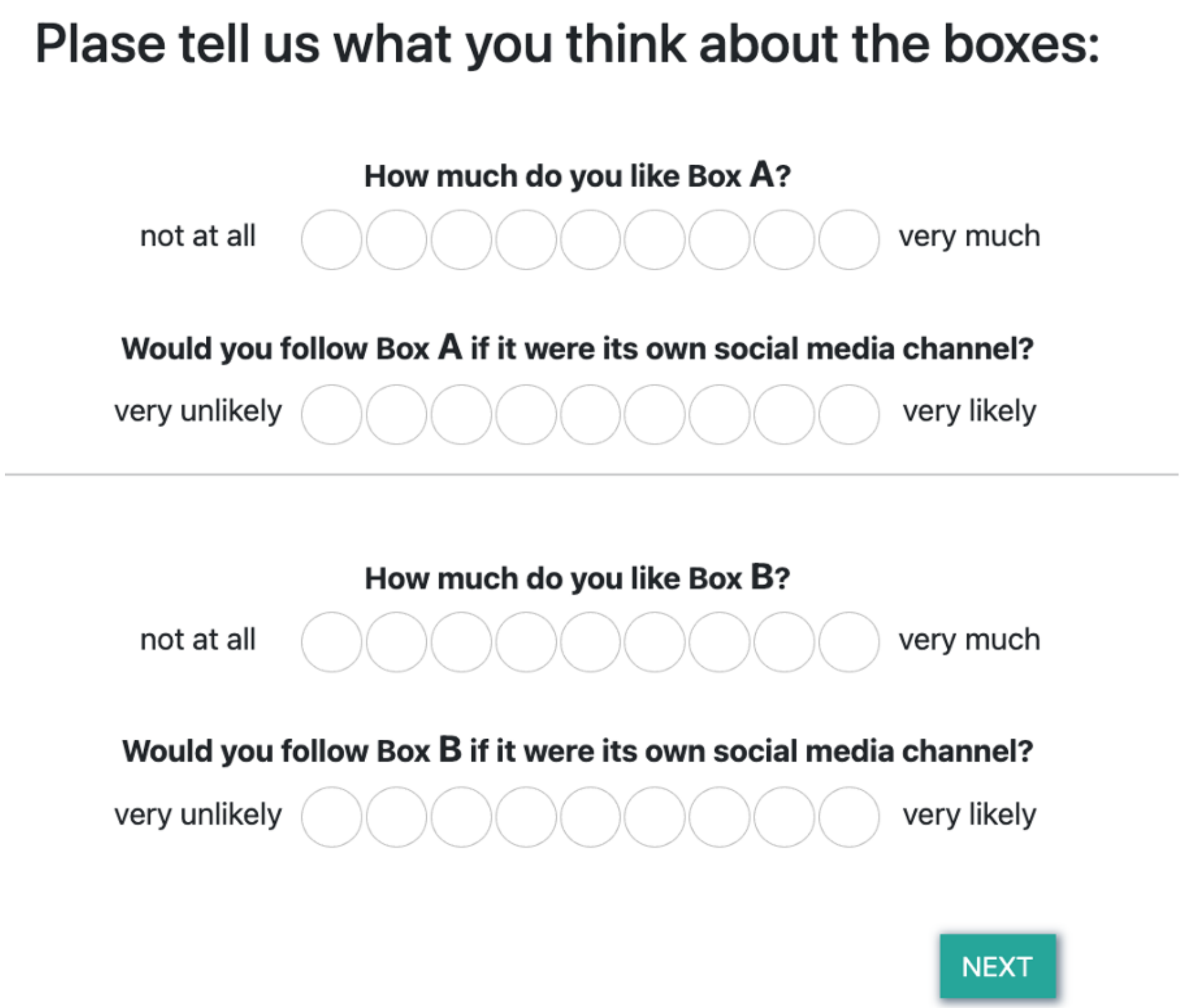

*Figure S*3. Screenshot showing questions to assess impression of the boxes every fifth round.

Additional Analyses

Distribution Plots for Initial Climate Change Concern

Figure S4 shows the distribution of initial climate change concern by country. As can be seen, the variance in initial concern is greater in Germany and the U.S. than in China, where most of the participants report climate concern above the midpoint. For Germany (Bimodality Coefficient = 0.64) and the U.S. (Bimodality Coefficient = 0.70), the bimodality coefficients are above the threshold of 0.56 indicating a potential bimodal distribution, for China (Bimodality Coefficient = 0.41) it is below this threshold.

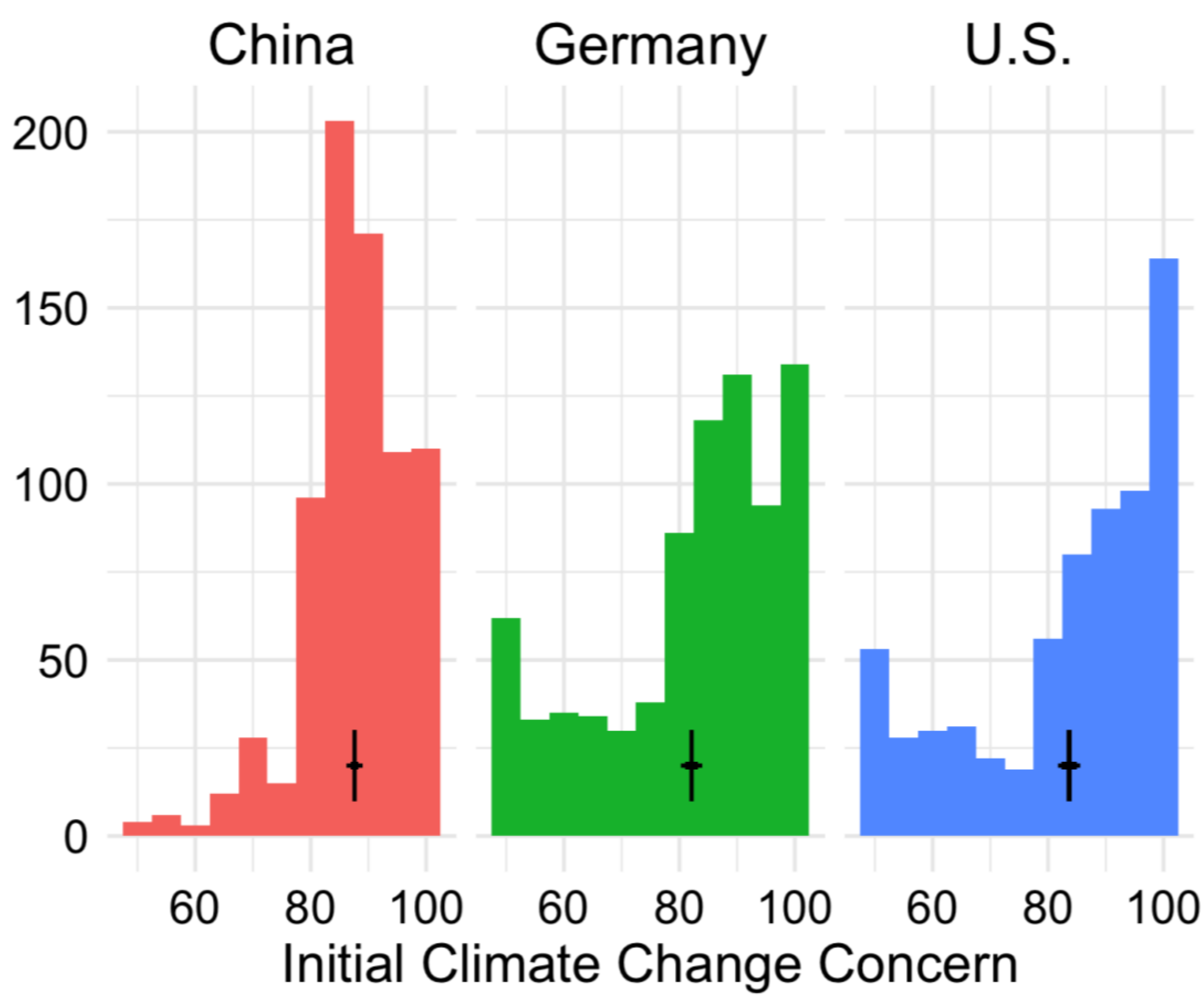

*Figure S*4. Histograms for Initial Climate Change concern by country. Black vertical line shows mean value, short vertical bars are standard errors for the mean.

Distribution Plots for Box Choices

Figure S5 shows the distribution of sum of Pro Climate Box Choices by country. Medium values were more frequent, especially in the Western countries, were the distribution was leptokurtic compared to a normal distribution.

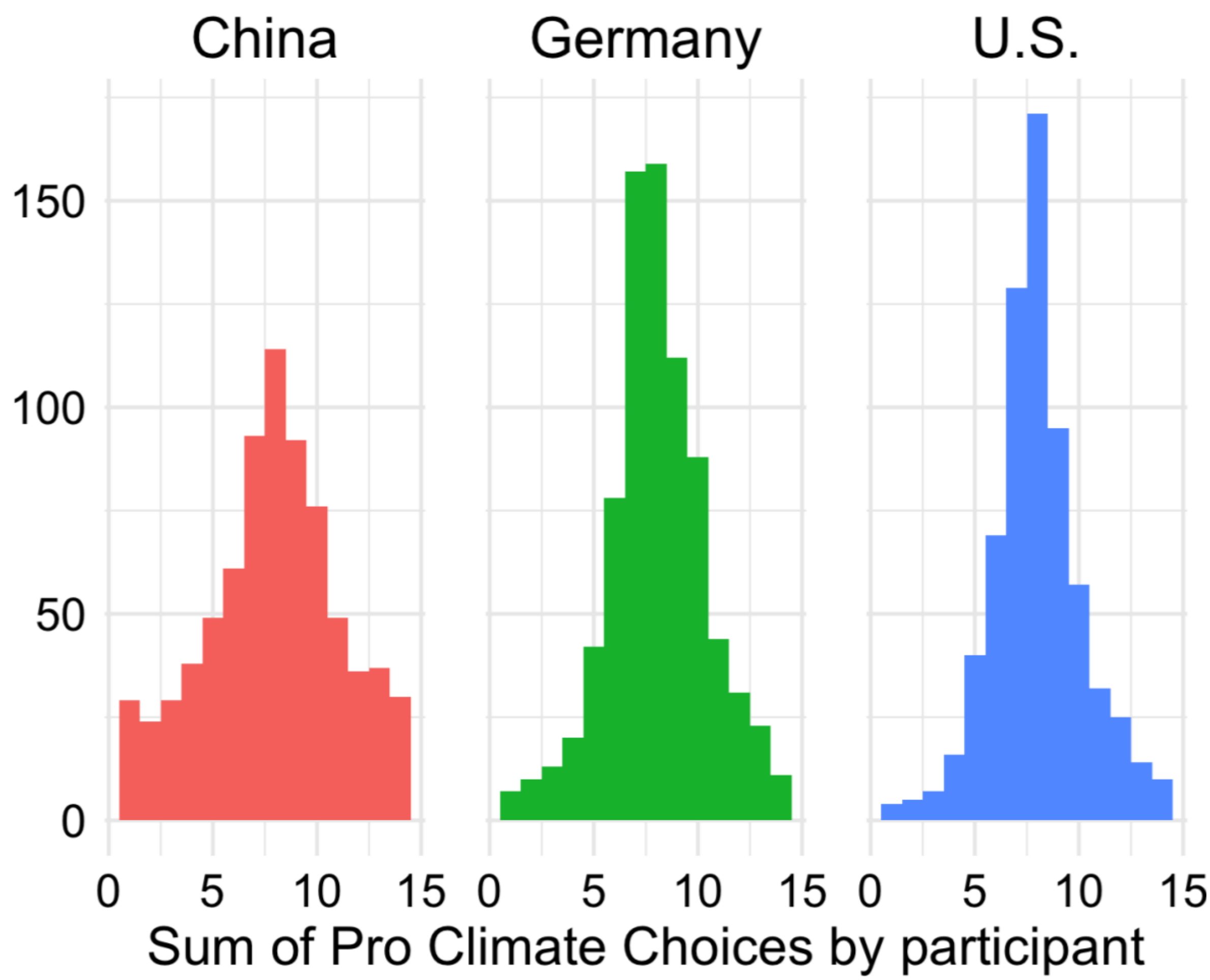

Figure S5. Histograms for Sum of Pro Climate Box Choices. Participants make 15 binary decisions between the Pro and Anti Climate Box.

Relationship between Political Orientation and Climate Change Views in the U.S. and Germany

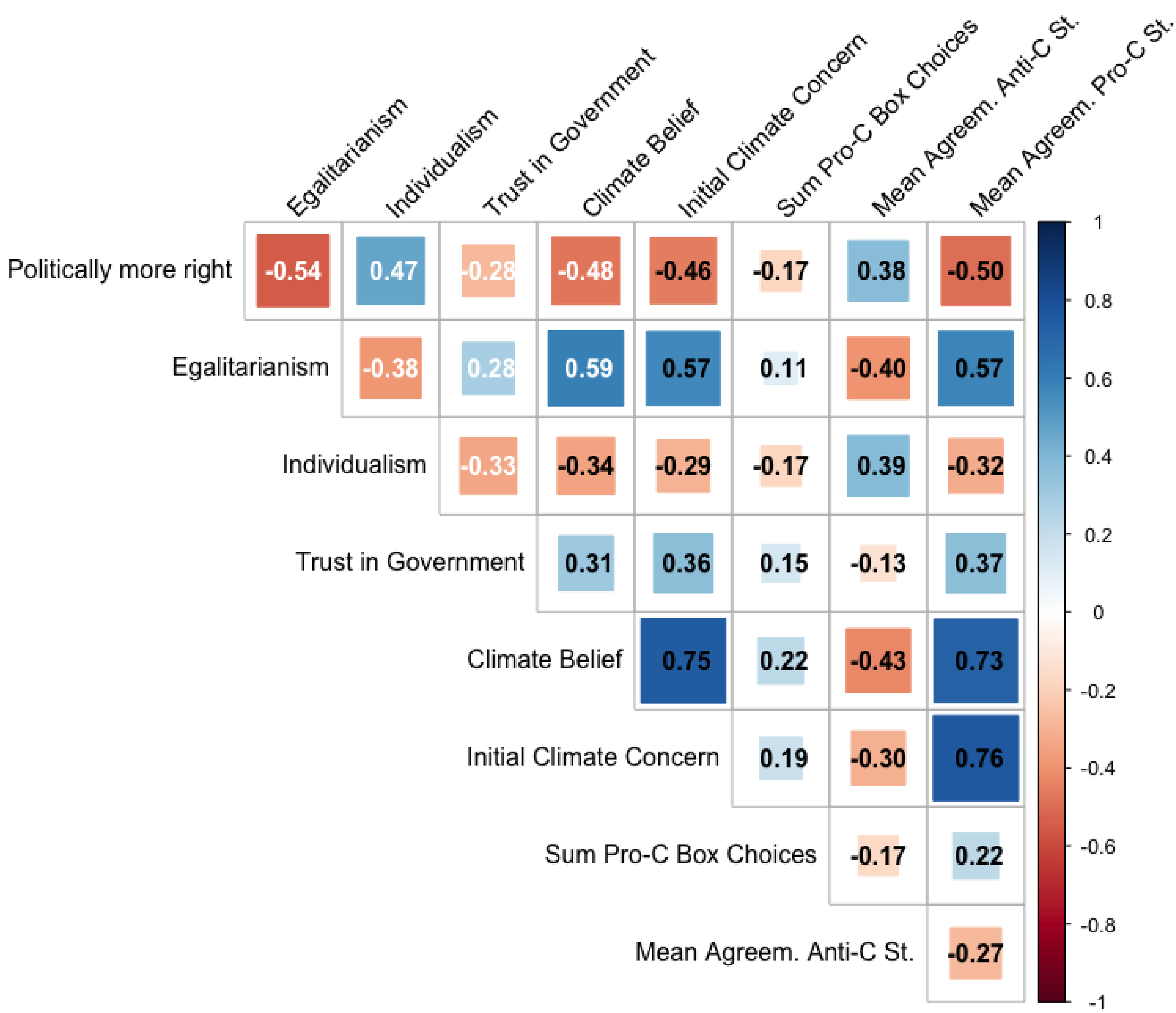

Figure S6. Correlation between Political and Climate Variables in the **United States.**
St = Statement; C = Climate

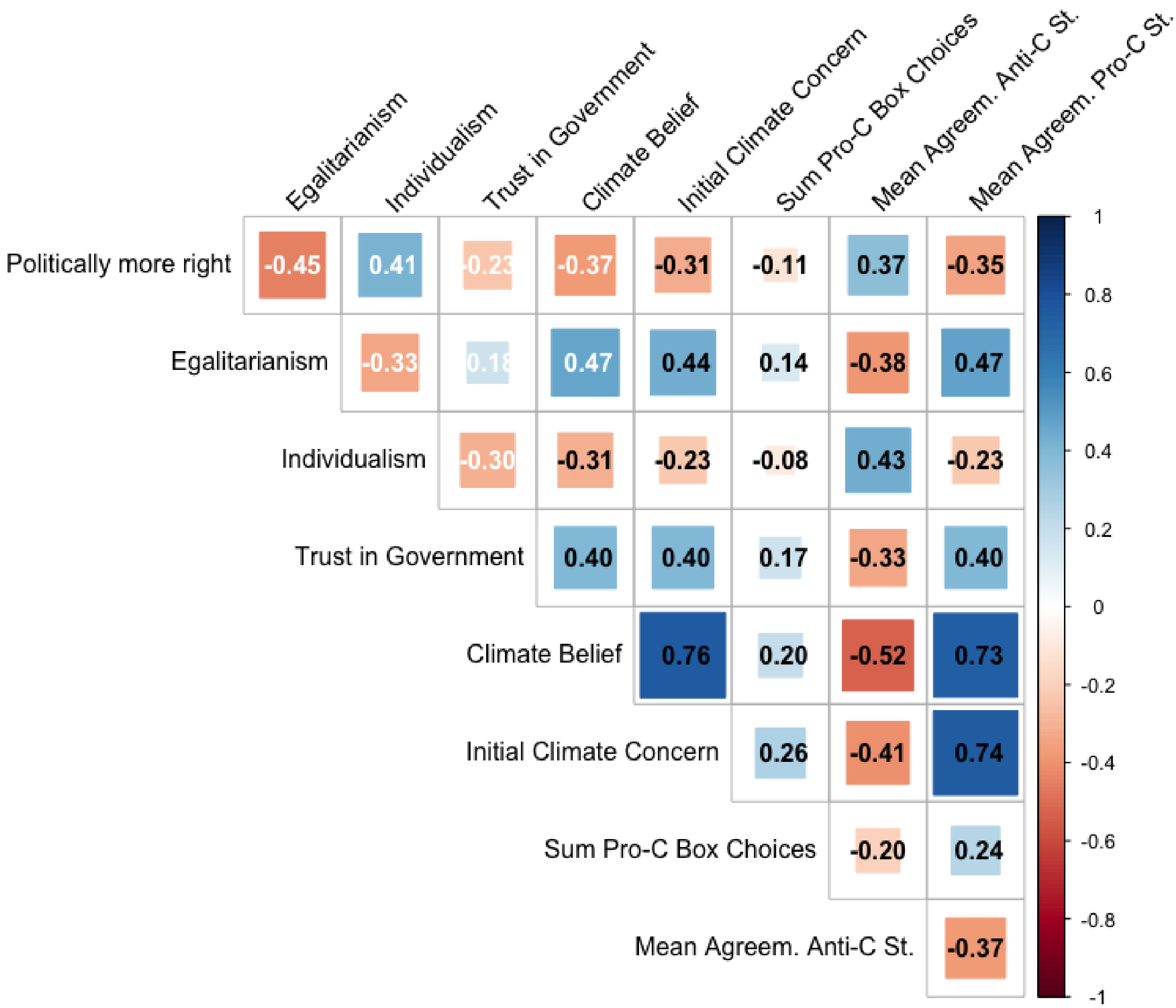

Figure S7. Correlation between Political and Climate Variables in **Germany.**
St = Statement; C = Climate

Table S5

Regression weights for logistic mixed effects models predicting box choices (1 = Pro-Climate Box) from participants' political orientation in the U.S. and in Germany.

	U.S.			Germany		
Predictors	*Odds Ratios*	*95%-CI*	*p*	*Odds Ratios*	*95%-CI*	*p*
(Intercept)	1.20	1.07 – 1.36	**0.002**	1.19	1.04 – 1.36	**0.009**
Political orientation (more left-wing)	1.11	1.06 – 1.16	**<0.001**	1.07	1.02 – 1.12	**0.006**
Round Number	1.05	1.01 – 1.09	**0.023**	1.10	1.06 – 1.14	**<0.001**
Box A is Anti Climate	0.90	0.82 – 0.98	**0.018**	0.95	0.86 – 1.04	0.247
Position Sampling Task	1.00	0.95 – 1.05	0.993	1.00	0.95 – 1.06	0.934
**Random Effects**						
σ^2	3.29			3.29		
τ_{00}	0.08 $_{participantID}$			0.15 $_{participantID}$		
ICC	0.02			0.04		
N	674 $_{participantID}$			795 $_{participantID}$		
Observations	10110			11925		
Marginal R^2 / Conditional R^2	0.004 / 0.027			0.004 / 0.048		

Plot visualizing the interaction between statement type and climate beliefs

Figure S6 shows the effects of the mixed effect regression model for confirmatory statement evaluation. Participants with higher initial climate beliefs agree more with Pro-climate statements and less with Anti-climate statements. The opposite pattern emerged for participants with lower initial climate beliefs. These patterns are similar for all countries. However, in China this effect is smaller, with less differences in agreement between participants with low and high climate beliefs, especially for Anti-climate statements.

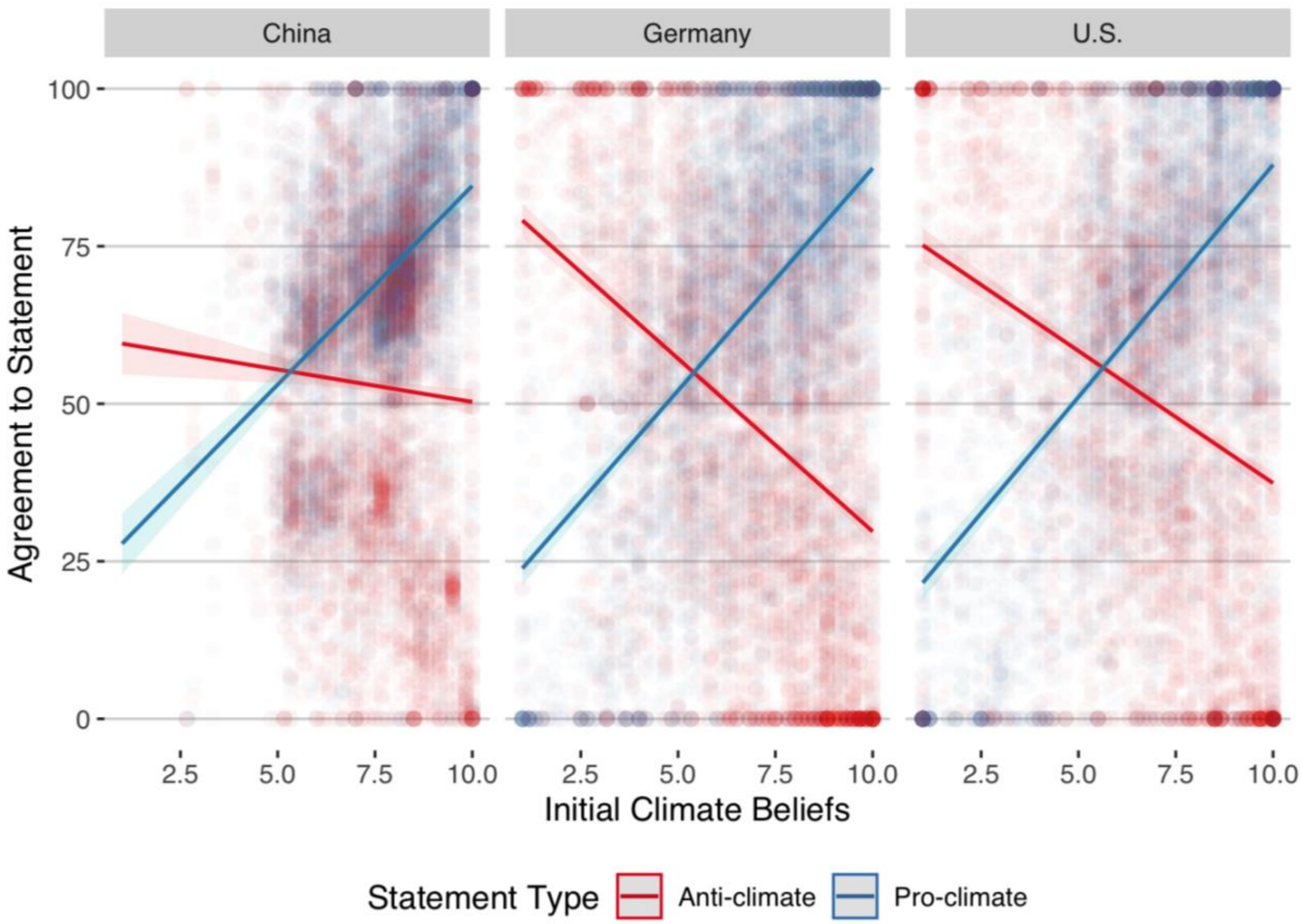

*Figure S*8. Predicted values of agreement to statement from mixed effect regression models by statement type (Pro- vs. Anti-climate) and initial climate beliefs, separately for each country.

Additional Hypotheses and Results

Table S6

Overview of Hypotheses reported in the manuscript and in the preregistration

Number in Manuscript	Number in Preregistration	Hypothesis Content
H1	H1	Confirmatory box choice
H1b	–	…with temporal dynamic
H1c	H1b	…with country differences
H1d	H4	Confirmatory statement evaluation
H1e	H4b	…with country differences
H2	H2	Effect of statements on concern
H2b	H2b	…moderated by agreement
H2c	–	…with country differences
H3	H2c	Reinforcing spiral
H3b	–	…with country differences
–	H3	Individual differences box rating
–	H3b	…with country differences
–	H5	Box switching *Not reported here, as part of a separate research project

Analysis of Pro- and Anti-climate Box Rating

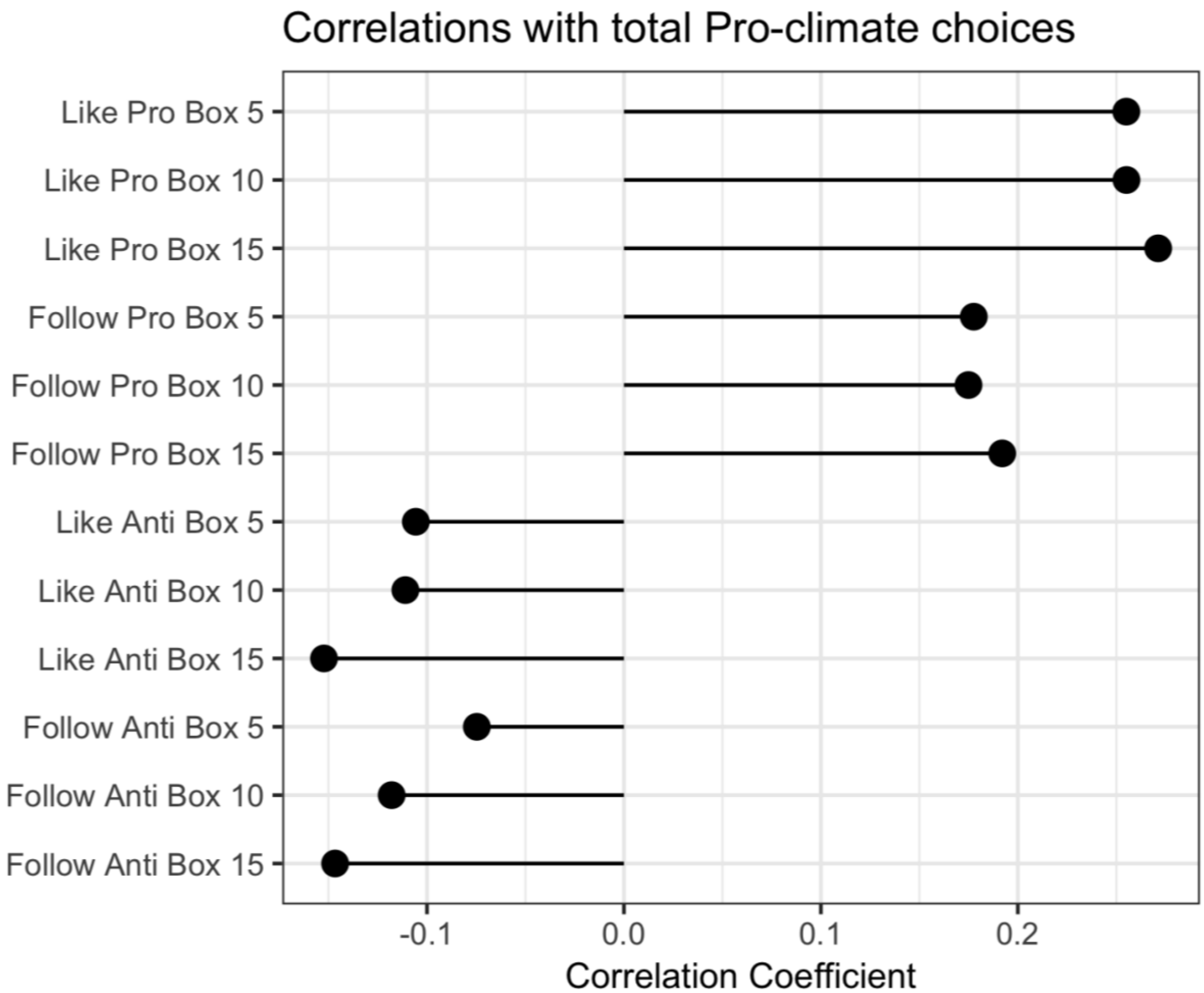

Figure S9. Plot displaying the correlation between the sum of Pro-Climate box choices and the rating of the Pro- and Anti-climate box.

In the preregistration, we expected that box ratings could be predicted by participants characteristics by their initial climate beliefs, their political orientation and their world views. Specifically, we expected higher initial beliefs, a more liberal (left-wing) orientation and more egalitarian and more communitarian worldviews to be associated with a greater liking for the Pro-Climate Box. Correspondingly, we expected lower initial climate beliefs, more conservative (right-wing) orientation, more hierarchical and more individualist worldviews to be associated with a greater liking for the Anti-Climate Box. Assessing political preferences in China is usually

not possible. We had planned to assess worldview variables (for egalitarian or hierarchical values) as proxies for political attitudes. However, given the sensitive nature of the worldview questionnaires, we were not able to measure worldviews in China. Therefore, for models including data from China, we only report the relationship with initial climate beliefs. Variables on political attitudes and worldviews are included in the country-specific models for the U.S. and Germany. Figure S8 and Figure S9 show the mean liking of the Pro- and Anti-Climate box in rounds 5, 10 and 15 for participants divided into quartiles of initial climate beliefs.

Table S7

Regression weights for linear mixed effects models predicting liking of the Pro- and Anti-climate box from participants' initial climate beliefs over time.

	**DV: Liking Pro-climate box**			**Liking Anti-climate box**		
Predictors	*Estimates*	*95%-CI*	*p*	*Estimates*	*95%-CI*	*p*
(Intercept)	6.37	6.21 – 6.53	**<0.001**	5.74	5.54 – 5.95	**<0.001**
Initial climate beliefs	0.88	0.81 – 0.95	**<0.001**	-0.38	-0.46 – -0.30	**<0.001**
Round number	0.05	0.02 – 0.08	**0.001**	-0.12	-0.15 – -0.09	**<0.001**
Country [Germany]	-0.41	-0.59 – -0.23	**<0.001**	-1.15	-1.37 – -0.92	**<0.001**
Country [U.S.]	-0.09	-0.28 – 0.10	0.363	-0.55	-0.79 – -0.31	**<0.001**
Position Sampling Task	-0.03	-0.13 – 0.06	0.481	-0.05	-0.16 – 0.07	0.425
Box A is Anti Climate	-0.03	-0.16 – 0.10	0.678	-0.16	-0.32 – 0.01	0.061
Initial beliefs × round number	0.11	0.08 – 0.14	**<0.001**	-0.15	-0.18 – -0.12	**<0.001**
Random Effects						
σ^2	1.44			1.64		
τ_{00}	1.97 $_{\text{participantID}}$			3.30 $_{\text{participantID}}$		
ICC	0.58			0.67		
N	2226 $_{\text{participantID}}$			2226 $_{\text{participantID}}$		
Observations	6678			6678		

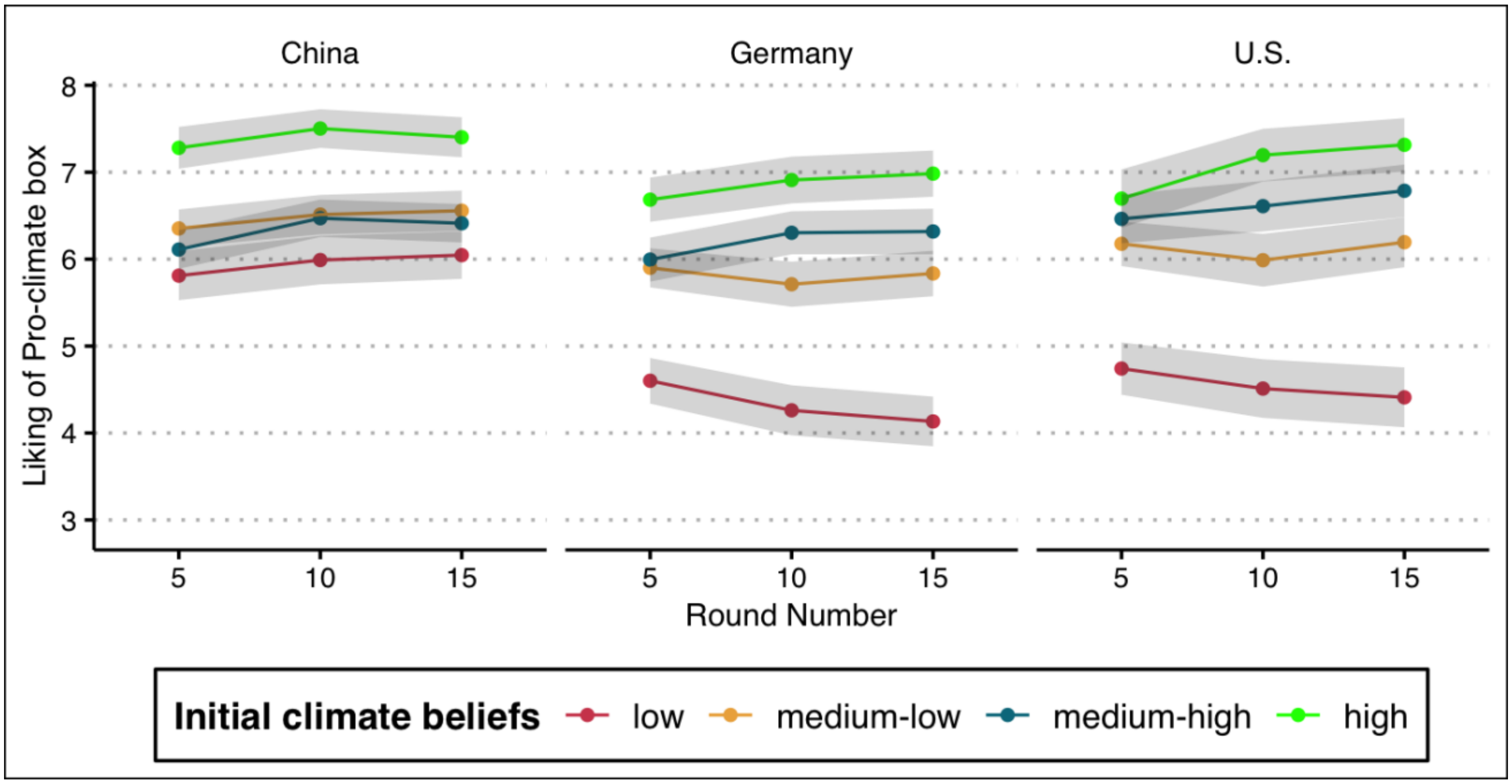

Figure S10. Plot displaying the mean liking of the **Pro**-climate box in three measurement points (after round 5, 10, and 15) by quartiles of initial climate belief and country. Error bands show 95%-Confidence intervals of the mean.

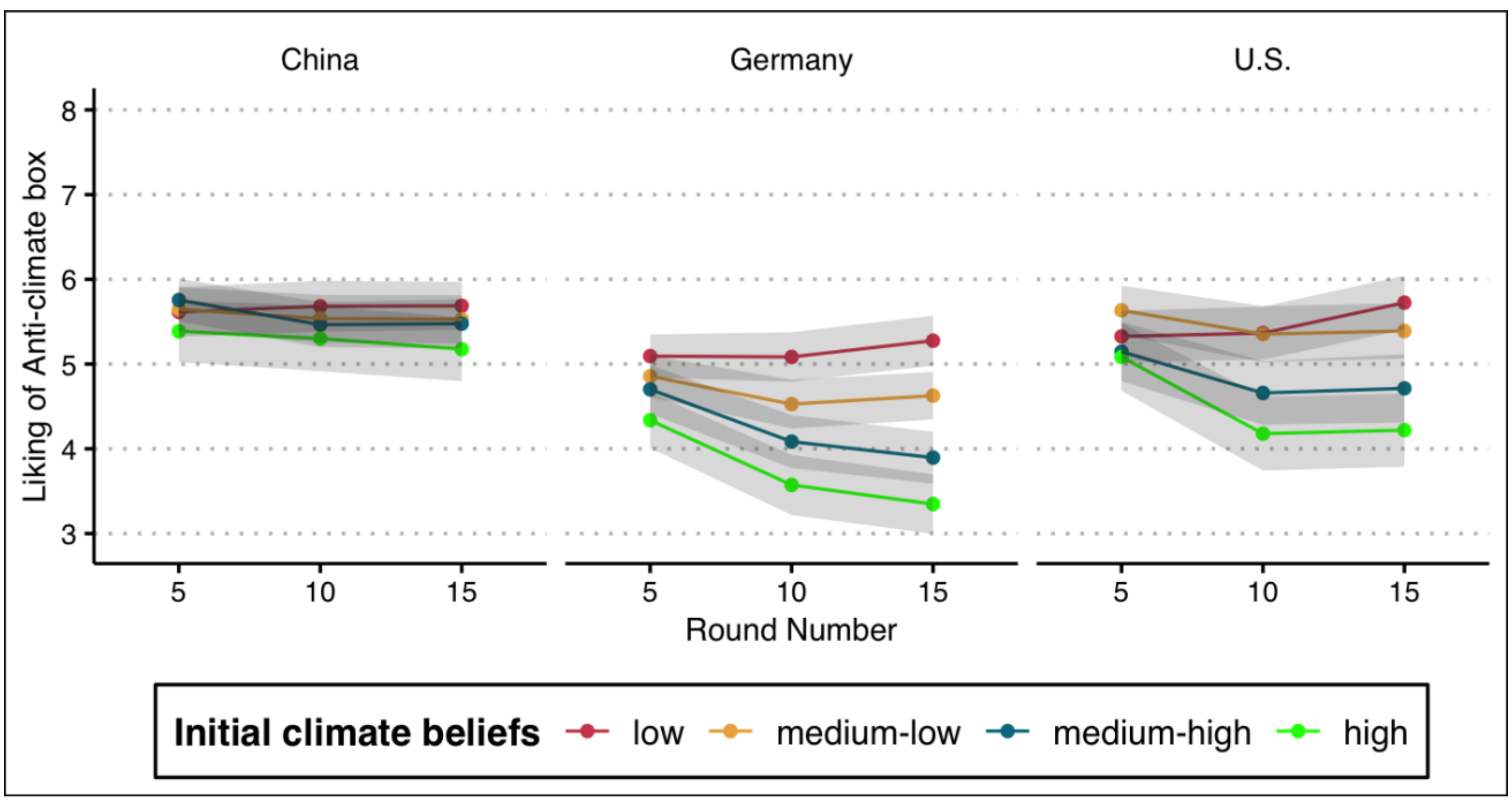

Figure S11. Plot displaying the mean liking of the **Anti**-climate box in three measurement points (after round 5, 10, and 15) by quartiles of initial climate belief and country. Error bands show 95%-Confidence intervals of the mean.

Table S8

Regression weights for linear mixed effects models predicting liking of the **Pro-**climate box from participants' initial climate beliefs and world views over time separately by country.

	China		U.S.		Germany	
Predictors	*Estimates [95%-CI]*	*p*	*Estimates [95%-CI]*	*p*	*Estimates [95%-CI]*	*p*
(Intercept)	6.68 [6.54 – 6.82]	**<0.001**	6.07 [5.74 – 6.39]	**<0.001**	5.84 [5.51 – 6.16]	**<0.001**
Initial climate beliefs	0.56 [0.46 – 0.65]	**<0.001**	0.86 [0.69 – 1.02]	**<0.001**	0.79 [0.66 – 0.93]	**<0.001**
Round number	0.09 [0.04 – 0.14]	**<0.001**	0.06 [0.00 – 0.12]	**0.048**	0.01 [-0.03 – 0.05]	0.737
Box A is Anti-climate	-0.28 [-0.48 – -0.08]	**0.006**	0.10 [-0.15 – 0.34]	0.436	0.06 [-0.17 – 0.29]	0.619
Initial beliefs × round number	0.00 [-0.05 – 0.05]	0.969	0.14 [0.08 – 0.20]	**<0.001**	0.14 [0.10 – 0.19]	**<0.001**
Position sampling task			-0.02 [-0.16 – 0.11]	0.720	-0.04 [-0.18 – 0.10]	0.562
Pol. orientation (more rightwing/conservative)			-0.17 [-0.32 – -0.01]	**0.033**	-0.09 [-0.22 – 0.05]	0.205
Egalitarian worldview			0.26 [0.09 – 0.44]	**0.003**	0.37 [0.23 – 0.50]	**<0.001**
Individualistic worldview			0.20 [0.06 – 0.35]	**0.006**	0.18 [0.05 – 0.31]	**0.007**
**Random Effects**						
σ^2	1.36		1.93		1.09	
τ_{00}	$1.46_{participantID}$		$1.95_{participantID}$		$2.29_{participantID}$	
ICC	0.52		0.50		0.68	
N	$757_{participantID}$		$674_{participantID}$		$795_{participantID}$	
Observations	2271		2022		2385	

Table S9

Regression weights for linear mixed effects models predicting liking of the **Anti**-climate box from participants' initial climate beliefs and world views over time separately by country.

	China		U.S.		Germany	
Predictors	*Estimates [95%-CI]*	*p*	*Estimates [95%-CI]*	*p*	*Estimates [95%-CI]*	*p*
(Intercept)	5.61 [5.41 – 5.80]	**<0.001**	5.16 [4.76 – 5.56]	**<0.001**	4.69 [4.32 – 5.06]	**<0.001**
Initial climate beliefs	-0.12 [-0.26 – 0.02]	0.082	-0.05 [-0.26 – 0.15]	0.621	-0.33 [-0.48 – -0.18]	**<0.001**
Round number	-0.05 [-0.10 – -0.00]	**0.032**	-0.11 [-0.18 – -0.05]	**0.001**	-0.18 [-0.23 – -0.14]	**<0.001**
Box A is Anti-climate	-0.16 [-0.43 – 0.11]	0.252	-0.15 [-0.45 – 0.16]	0.346	-0.21 [-0.47 – 0.05]	0.108
Initial beliefs × round number	-0.06 [-0.11 – -0.01]	**0.020**	-0.19 [-0.26 – -0.13]	**<0.001**	-0.20 [-0.24 – -0.15]	**<0.001**
Position sampling task			-0.01 [-0.17 – 0.16]	0.914	-0.06 [-0.22 – 0.10]	0.465
Pol. orientation (more rightwing/conservative)			0.31 [0.12 – 0.50]	**0.002**	0.14 [-0.01 – 0.29]	0.069
Egalitarian worldview			-0.06 [-0.28 – 0.15]	0.571	-0.01 [-0.16 – 0.15]	0.940
Individualistic worldview			0.36 [0.18 – 0.53]	**<0.001**	0.48 [0.43 – 0.63]	**<0.001**
**Random Effects**						
σ^2	1.37		2.21		1.38	
τ_{00}	$3.23_{\text{participantID}}$		$3.21_{\text{participantID}}$		$2.97_{\text{participantID}}$	
ICC	0.70		0.59		0.68	
N	$757_{\text{participantID}}$		$674_{\text{participantID}}$		$795_{\text{participantID}}$	
Observations	2271		2022		2385	

Robustness Analysis Including Inattentive Participants

We repeated the analyses reported in the manuscript with the full participant sample (including participants previously excluded for inattentiveness) as a robustness check. The full dataset includes 379 participants from China, 88 participants from Germany and 206 participants from the U.S. that were previously excluded because they failed to pass at least one of the attention checks. The full dataset contains data from 2899 participants, 1136 from China, 883 from Germany and 880 from the U.S.

Hypothesis 1: Confirmatory Information Sampling (robustness check)

In line with the findings reported in the manuscript for the subset of attentive participants, we find a significant effect of initial climate beliefs on box choice when using the full sample including participants who failed the attention checks (see Table S10).

Table S10

Odds ratios for logistic mixed effects models predicting box choice box for all participants (including those that failed attention checks).

	Simple Model			Model with temporal interaction		
Predictors	*Estimates*	*95%-CI*	*p*	*Estimates*	*95%-CI*	*p*
(Intercept)	1.39	1.28 – 1.52	**<0.001**	1.39	1.28 – 1.52	**<0.0**
Initial climate beliefs	1.12	1.08 – 1.16	**<0.001**	1.12	1.08 – 1.16	**<0.001**
Round number	1.08	1.05 – 1.10	**<0.001**	1.08	1.05 – 1.10	**<0.001**
Country [Ger.]	1.11	1.00 – 1.23	**0.047**	1.11	1.00 – 1.23	**0.047**
Country [U.S.]	1.03	0.93 – 1.15	0.567	1.03	0.93 – 1.15	0.565
Position Sampling Task	0.99	0.94 – 1.05	0.835	0.99	0.94 – 1.05	0.836
Box A is Anti Climate	0.63	0.58 – 0.67	**<0.001**	0.63	0.58 – 0.67	**<0.001**
Initial beliefs × round number				1.06	1.04 – 1.08	**<0.001**
Random Effects						
σ^2	3.29			3.29		
τ_{00}	0.67 $_{\text{participantID}}$			0.68 $_{\text{participantID}}$		
ICC	0.17			0.17		
N	2899 $_{\text{participantID}}$			2899 $_{\text{participantID}}$		
Observations	43485			43485		

In Table S11, results of mixed effects regression models by country are shown. The preregistered hypothesis that box choice can be predicted from participants initial beliefs is significant in every country and results are similar to the models reported in the manuscript with attentive participants only. There is one difference in the effect for counterbalancing of boxes (Box A being the Pro- or Anti-climate box) showing that when inattentive participants are included, the effect of box allocation is significant in all countries. With only attentive participants, the effect is only significant in China and with a smaller effect size. This means that with inattentive participants, participants showed a preference for choosing the Box A on the left-hand side irrespective of its content. Position of the task within the panel (not applicable in China) remained irrelevant for box choice behavior.

Table S11

Odds ratios for logistic mixed effects models predicting box choice box for all participants separately by country (including those that failed attention checks).

	**China**		**U.S.**		**Germany**	
Predictors	*Estimates [95%-CI]*	*p*	*Estimates [95%-CI]*	*p*	*Estimates [95%-CI]*	*p*
(Intercept)	1.96 [1.77 – 2.20]	**<0.001**	1.19 [1.04 – 1.37]	**0.014**	1.25 [1.08 – 1.44]	**0.002**
Initial climate beliefs	1.10 [1.02 – 1.18]	**0.019**	1.12 [1.06 – 1.18]	**<0.001**	1.14 [1.08 – 1.19]	**<0.001**
Round number	1.10 [1.07 – 1.14]	**<0.001**	1.03 [1.00 – 1.07]	0.088	1.09 [1.05 – 1.13]	**<0.001**
Box A is Anti-climate	0.33 [0.28 – 0.39]	**<0.001**	0.87 [0.79 – 0.97]	**0.010**	0.89 [0.81 – 0.99]	**0.025**
Position sampling task			1.00 [0.94 – 1.06]	0.918	1.00 [0.94 – 1.06]	0.979
**Random Effects**						
σ^2	3.29		3.29		3.29	
τ_{00}	1.34 $_{participantID}$		0.33 $_{participantID}$		0.28 $_{participantID}$	
ICC	0.29		0.09		0.08	
N	1136 $_{participantID}$		880 $_{participantID}$		883 $_{participantID}$	
Observations	17040		13200		13245	

Hypothesis 1d: Confirmatory Statement Evaluation (robustness check)

In line with the findings from the manuscript and confirming our preregistered hypothesis, we find that the evaluation of the climate-related statements can be predicted from participants initial climate beliefs. Participants with higher initial climate beliefs agree more with Pro-climate statements compared to those with lower initial climate beliefs and vice versa for Anti-climate statements. There are also differences between countries in the strength of this effect, as indicated by the significant three-way interaction between statement type, initial climate beliefs and country. The results suggests that confirmatory statement evaluation (i.e. agreeing with a statement more if it aligns with one's initial climate beliefs) is stronger in Western countries than in China (see Table S12 for full regression results).

Table S12

Regression weights for linear mixed effects models predicting agreement with statement after reading a Pro- or Anti-climate statement.

	Simple Model			Model with country-interactions		
Predictors	*Estimates*	*95%-CI*	*p*	*Estimates*	*95%-CI*	*p*
(Intercept)	54.89	53.39 – 56.39	**<0.001**	59.21	57.54 – 60.88	**<0.001**
Statement type [Pro-Climate]	16.21	15.63 – 16.79	**<0.001**	9.73	8.30 – 11.16	**<0.001**
Initial climate beliefs	-7.16	-7.71 – -6.60	**<0.001**	-2.32	-3.62 – -1.02	**<0.001**
Country [Germany]	-3.22	-4.68 – -1.76	**<0.001**	-12.18	-14.14 – -10.22	**<0.001**
Country [U.S.]	0.29	-1.22 – 1.81	0.704	-5.05	-7.05 – -3.04	**<0.001**
Round number	0.00	-0.05 – 0.05	0.930	0.00	-0.05 – 0.05	0.997
Box A is Anti-climate	-0.14	-1.14 – 0.87	0.791	-0.36	-1.35 – 0.64	0.482
Position sampling task	-0.36	-1.11 – 0.38	0.334	-0.41	-1.14 – 0.32	0.271
St. type [Pro-Climate] × Initial beliefs	20.68	20.23 – 21.14	**<0.001**	13.18	12.08 – 14.29	**<0.001**
St. type [Pro-Climate] × [Germany]				11.02	9.35 – 12.68	**<0.001**
St. type [Pro-Climate] × [U.S.]				4.94	3.28 – 6.61	**<0.001**
Initial beliefs × [Germany]				-7.98	-9.55 – -6.41	**<0.001**
Initial beliefs × [U.S.]				-4.11	-5.66 – -2.56	**<0.001**
St. type [Pro-Climate] × initial beliefs × [Germany]				11.02	9.70 – 12.34	**<0.001**
St. type [Pro-Climate] × initial beliefs × [U.S.]				7.60	6.30 – 8.90	**<0.001**
**Random Effects**						
σ^2	487.97			483.53		
τ_{00}	155.97 participantID			151.78 participantID		
	28.55 statementID			20.12 statementID		
ICC	0.27			0.26		
N	2899 participantID			2899 participantID		
	219 statementID			219 statementID		
Observations	43485			43485		

Hypothesis 2: Influences of Information Sampling on Climate Concern (robustness check)

In line with the findings from the manuscript and confirming our preregistered hypotheses, we find that immediately after reading a Pro-[Anti-]climate statement, participants reported higher [lower] climate change concern compared to their initial concern (cf. Table S13).

Table S13

Regression weights for linear mixed effects models predicting climate change concern after reading a Pro- or Anti-climate statement.

	**Simple Model**			**Model with Interaction by agreement to statement**		
Predictors	*Estimates*	*95%-CI*	*p*	*Estimates*	*95%-CI*	*p*
(Intercept)	11.36	9.63 – 13.10	**<0.001**	12.31	10.59 – 14.02	**<0.001**
Statement type [Anti-climate]	-1.39	-1.61 – -1.17	**<0.001**	-0.56	-0.78 – -0.34	**<0.001**
Country [Germany]	0.03	-1.26 – 1.32	0.968	0.17	-1.10 – 1.44	0.795
Country [U.S.]	-0.96	-2.29 – 0.37	0.158	-0.85	-2.16 – 0.46	0.204
Round number	0.01	-0.01 – 0.03	0.377	0.00	-0.02 – 0.03	0.668
Initial Climate concern	0.84	0.82 – 0.86	**<0.001**	0.82	0.80 – 0.84	**<0.001**
Position sampling task	-0.07	-0.74 – 0.61	0.849	-0.06	-0.72 – 0.61	0.866
Box A is Anti-climate	1.32	0.41 – 2.23	**0.004**	1.29	0.39 – 2.18	**0.005**
Agreement				2.64	2.46 – 2.81	**<0.001**
St. type [Anti-climate] × Agreement				-1.54	-1.77 – -1.30	**<0.001**
**Random Effects**						
σ^2	102.35			99.95		
τ_{00}	148.91 $_{participantID}$			144.25 $_{participantID}$		
	0.24 $_{statementID}$			0.11 $_{statementID}$		
ICC	0.59			0.59		
N	2899 $_{participantID}$			2899 $_{participantID}$		
	219 $_{statementID}$			219 $_{statementID}$		
Observations	43485			43485		

Hypothesis 3: Reinforcing spirals model (robustness check)

In line with the findings from the manuscript and confirming our preregistered hypotheses, we find that participants' changes in climate change concern over time can be predicted from the total number of Pro-climate box choices in the U.S. This effect was not significant in China or in Germany. (see Table S14 for regression results).

Table S14

Regression weights for linear mixed effects models predicting trends in climate change concern depending on total number of Pro-climate box choices.

	**U.S.**		**China**		**Germany**	
Predictors	*Estimates*	*p*	*Estimates*	*p*	*Estimates*	*p*
(Intercept)	0.04 [0.33 – 0.40]	0.849	-0.05 [0.14 – 0.05]	0.343	0.25 [-0.05 – 0.54]	0.101
Sum Pro-climate choices	0.20 [0.07 – 0.34]	**0.003**	0.03 [0.04 – 0.10]	0.354	0.07 [-0.04 – 0.17]	0.226
Initial climate concern	-0.06 [-0.19 – 0.07]	0.380	-0.26 [-0.33 – -0.20]	**<0.001**	-0.12 [-0.23 – -0.02]	**0.022**
Box A is Anti-climate	0.26 [-0.01 – 0.53]	0.056	0.11 [0.03 – 0.25]	0.113	0.03 [-0.18 – 0.24]	0.766
Position sampling task	0.00 [-0.16 – 0.16]	0.993			-0.00 [-0.13 – 0.12]	0.956
Observations	880		1136		695	
R^2 / R^2 adjusted	0.013 / 0.009		0.057 / 0.055		0.013 / 0.007	

Software used for statistical data analysis

We used R version 4.4.0 (R Core Team, 2024) and the following R packages: broom.mixed v. 0.2.9.6 (Bolker & Robinson, 2024), corrplot v. 0.95 (Wei & Simko, 2024), fabricatr v. 1.0.2 (Blair et al., 2024), ggthemes v. 5.1.0 (Arnold, 2024), grateful v. 0.2.10 (Rodriguez-Sanchez & Jackson, 2023), here v. 1.0.1 (Müller, 2020), kableExtra v. 1.4.0 (Zhu, 2024), knitr v. 1.49 (Xie, 2014, 2015, 2024), lme4 v. 1.1.36 (Bates, Mächler, Bolker, & Walker, 2015), lmerTest v. 3.1.3 (Kuznetsova, Brockhoff, & Christensen, 2017), mousetrap v. 3.2.3 (Wulff, Kieslich, Henninger, Haslbeck, & Schulte-Mecklenbeck, 2023), papaja v. 0.1.3 (Aust & Barth, 2024), patchwork v. 1.3.0 (Pedersen, 2024), rmarkdown v. 2.29 (Allaire et al., 2024; Xie, Allaire, & Grolemund, 2018; Xie, Dervieux, & Riederer, 2020), scales v. 1.3.0 (Wickham, Pedersen, & Seidel, 2023), sjPlot v. 2.8.17 (Lüdecke, 2024), table1 v. 1.4.3 (Rich, 2023), tidyverse v. 2.0.0 (Wickham et al., 2019), running in RStudio v. 2024.12.0.467 (Posit team, 2024).

References

Allaire, J., Xie, Y., Dervieux, C., McPherson, J., Luraschi, J., Ushey, K., … Iannone, R. (2024). *rmarkdown: Dynamic documents for r*. Retrieved from https://github.com/rstudio/rmarkdown

Arnold, J. B. (2024). *ggthemes: Extra themes, scales and geoms for "ggplot2"*. Retrieved from https://CRAN.R-project.org/package=ggthemes

Aust, F., & Barth, M. (2024). *papaja: Prepare reproducible APA journal articles with R Markdown*. https://doi.org/10.32614/CRAN.package.papaja

Bates, D., Mächler, M., Bolker, B., & Walker, S. (2015). Fitting linear mixed-effects models using lme4. *Journal of Statistical Software*, *67*(1), 1–48. https://doi.org/10.18637/jss.v067.i01

Blair, G., Cooper, J., Coppock, A., Humphreys, M., Rudkin, A., & Fultz, N. (2024). *fabricatr: Imagine your data before you collect it*. Retrieved from https://CRAN.R-project.org/package=fabricatr

Bolker, B., & Robinson, D. (2024). *broom.mixed: Tidying methods for mixed models*. Retrieved from https://CRAN.R-project.org/package=broom.mixed

Kahan, D. M., Braman, D., Gastil, J., Slovic, P., & Mertz, C. K. (2007). Culture and Identity-Protective Cognition: Explaining the White-Male Effect in Risk Perception. *Journal of Empirical Legal Studies*, *4*(3), 465–505. https://doi.org/10.1111/j.1740-1461.2007.00097.x

Kuznetsova, A., Brockhoff, P. B., & Christensen, R. H. B. (2017). lmerTest package: Tests in linear mixed effects models. *Journal of Statistical Software*, *82*(13), 1–26. https://doi.org/10.18637/jss.v082.i13

Lüdecke, D. (2024). *sjPlot: Data visualization for statistics in social science*. Retrieved from https://CRAN.R-project.org/package=sjPlot

Müller, K. (2020). *here: A simpler way to find your files*. Retrieved from https://CRAN.R-project.org/package=here

Pedersen, T. L. (2024). *patchwork: The composer of plots*. Retrieved from https://CRAN.R-project.org/package=patchwork

Posit team. (2024). *RStudio: Integrated development environment for r*. Boston, MA: Posit Software, PBC. Retrieved from http://www.posit.co/

R Core Team. (2024). *R: A language and environment for statistical computing*. Vienna, Austria: R Foundation for Statistical Computing. Retrieved from https://www.R-project.org/

Rich, B. (2023). *table1: Tables of descriptive statistics in HTML*. Retrieved from https://CRAN.R-project.org/package=table1

Rodriguez-Sanchez, F., & Jackson, C. P. (2023). *grateful: Facilitate citation of r packages*. Retrieved from https://pakillo.github.io/grateful/

Truelove, H. B., & Greenberg, M. (2013). Who has become more open to nuclear power because of climate change? *Climatic Change*, *116*(2), 389–409. https://doi.org/10.1007/s10584-012-0497-2

van Valkengoed, A. M., Steg, L., & Perlaviciute, G. (2021). Development and validation of a climate change perceptions scale. *Journal of Environmental Psychology*, *76*, 101652. https://doi.org/10.1016/j.jenvp.2021.101652

Wei, T., & Simko, V. (2024). *R package "corrplot": Visualization of a correlation matrix*. Retrieved from https://github.com/taiyun/corrplot

Wickham, H., Averick, M., Bryan, J., Chang, W., McGowan, L. D., François, R., … Yutani, H. (2019). Welcome to the tidyverse. *Journal of Open Source Software*, *4*(43), 1686. https://doi.org/10.21105/joss.01686

Wickham, H., Pedersen, T. L., & Seidel, D. (2023). *scales: Scale functions for visualization*. Retrieved from https://CRAN.R-project.org/package=scales

Wulff, D. U., Kieslich, P. J., Henninger, F., Haslbeck, J. M. B., & Schulte-Mecklenbeck, M. (2023). *Movement tracking of psychological processes: A tutorial using mousetrap*. PsyArXiv. https://doi.org/10.31234/osf.io/v685r

Xie, Y. (2014). knitr: A comprehensive tool for reproducible research in R. In V. Stodden, F. Leisch, & R. D. Peng (Eds.), *Implementing reproducible computational research*. Chapman; Hall/CRC.

Xie, Y. (2015). *Dynamic documents with R and knitr* (2nd ed.). Boca Raton, Florida: Chapman; Hall/CRC. Retrieved from https://yihui.org/knitr/

Xie, Y. (2024). *knitr: A general-purpose package for dynamic report generation in r*. Retrieved from https://yihui.org/knitr/

Xie, Y., Allaire, J. J., & Grolemund, G. (2018). *R markdown: The definitive guide*. Boca Raton, Florida: Chapman; Hall/CRC. Retrieved from https://bookdown.org/yihui/rmarkdown

Xie, Y., Dervieux, C., & Riederer, E. (2020). *R markdown cookbook*. Boca Raton, Florida: Chapman; Hall/CRC. Retrieved from https://bookdown.org/yihui/rmarkdown-cookbook

Zhu, H. (2024). *kableExtra: Construct complex table with "kable" and pipe syntax*. Retrieved from https://CRAN.R-project.org/package=kableExtra
